# 1,2- and 1,1-Migratory Insertion Reactions of Silylated Germylene Adducts

**DOI:** 10.3390/molecules25030686

**Published:** 2020-02-06

**Authors:** Małgorzata Walewska, Judith Baumgartner, Christoph Marschner

**Affiliations:** Institut für Anorganische Chemie, Technische Universität Graz, Stremayrgasse 9, A-8010 Graz, Austria; m.walewska@gmx.at

**Keywords:** germylene, germanium dichloride, 1,1-insertion, 1,2-insertion

## Abstract

The reactions of the PMe_3_ adduct of the silylated germylene [(Me_3_Si)_3_Si]_2_Ge: with GeCl_2_·dioxane were found to yield 1,1-migratory insertion products of GeCl_2_ into one or two Ge–Si bonds. In a related reaction, a germylene was inserted with tris(trimethylsilyl)silyl and vinyl substituents into a Ge–Cl bond of GeCl_2_. This was followed by intramolecular trimethylsilyl chloride elimination to another cyclic germylene PMe_3_ adduct. The reaction of the GeCl_2_ mono-insertion product (Me_3_Si)_3_SiGe:GeCl_2_Si(SiMe_3_)_3_ with Me_3_SiC≡CH gave a mixture of alkyne mono- and diinsertion products. While the reaction of a divinylgermylene with GeCl_2_·dioxane only results in the exchange of the dioxane of GeCl_2_ against the divinylgermylene as base, the reaction of [(Me_3_Si)_3_Si]_2_Ge: with one GeCl_2_·dioxane and three phenylacetylenes gives a trivinylated germane with a chlorogermylene attached to one of the vinyl units.

## 1. Introduction

Low valent main group compounds such as carbenes and their higher congeners have attracted much attention due to their transition metal-like behavior [1,2,3,4]. While most of this similarity refers to the oxidative addition and reductive elimination processes, other elementary reaction steps typical for transition metals are also possible.

**Scheme 1:**
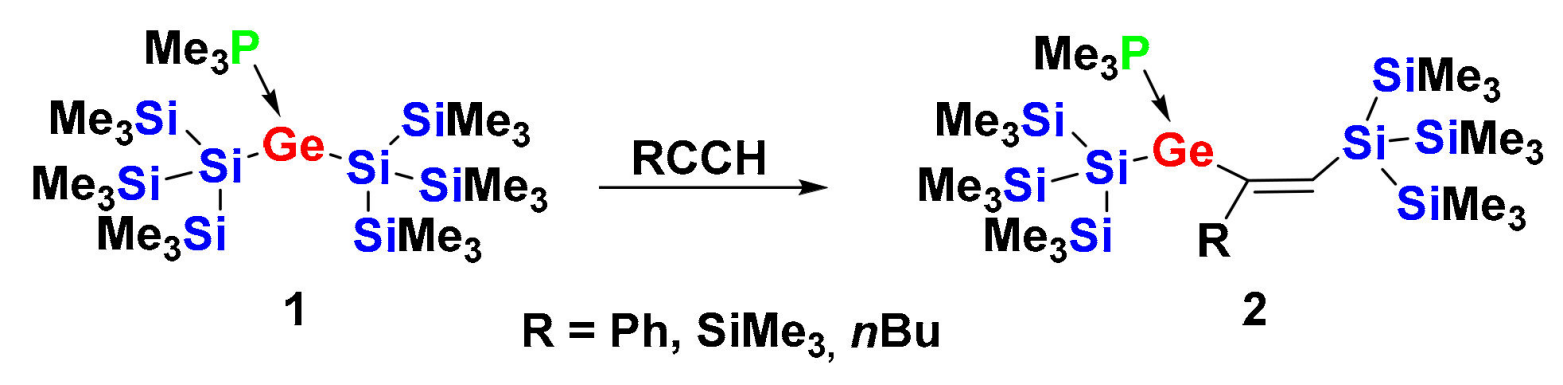
1,2-Migratory insertion reaction of alkynes into the Ge–Si bond of the silylated germylene PMe_3_ adduct **1**.

Recently, we reported the synthesis of the PMe_3_ adduct of bis[tris(trimethylsilyl)silyl]germylene (**1**) [5]. A most unusual reaction of this compound, we observed the regioselective 1,2-migratory insertion of phenylacetylene into one or two of the Ge–Si bonds of **1** (Scheme 1) [5]. While, typically, reactions of silylenes and germylenes with alkynes lead to oxidation and the formation of tetravalent sila- and germacyclopropenes [6,7,8,9,10,11,12], the observed phenylacetylene insertion case benefits from the fact that formation of the vinylgermylene PMe_3_ adduct (**2**) is thermodynamically favored compared to the respective germacyclopropene, which exists as an intermediate on the reaction pathway [13].

The 1,2-migratory insertion is not restricted to phenylacetylene. The same reaction could be also carried out with trimethylsilylactylene and 1-hexyne (Scheme 1) [13]. When phosphane adducts of cyclic disilylated germylenes were subjected to reactions with alkynes spirocyclic, vinylgermanes were obtained [14]. Similar alkyne insertion reactivity was later observed for a diborylstannylene [15]. A related ethylene insertion was observed previously in the Si–Sn bond of a stannylated silylene adduct [16], and later in the Si–Si bond of the stable acyclic silylene dipp(Me_3_Si)N-Si-Si(SiMe_3_)_3_ (dipp = 2,6-*i*Pr_2_C_6_H_3_) [17]. More recently, Sasamori, Tokitoh and co-workers reported the spectacular example of a digermyne acting as a main group catalyst for the cyclotrimerization of arylacetylenes. The key steps of the catalytic cycle are 1,2-migratory insertion reactions of arylacetylenes into Ge–C bonds of vinylgermylenes [18]. In the current account, we present the chemistry of **1** and related compounds with GeCl_2_·dioxane.

## 2. Results and Discussion

**Scheme 2:**
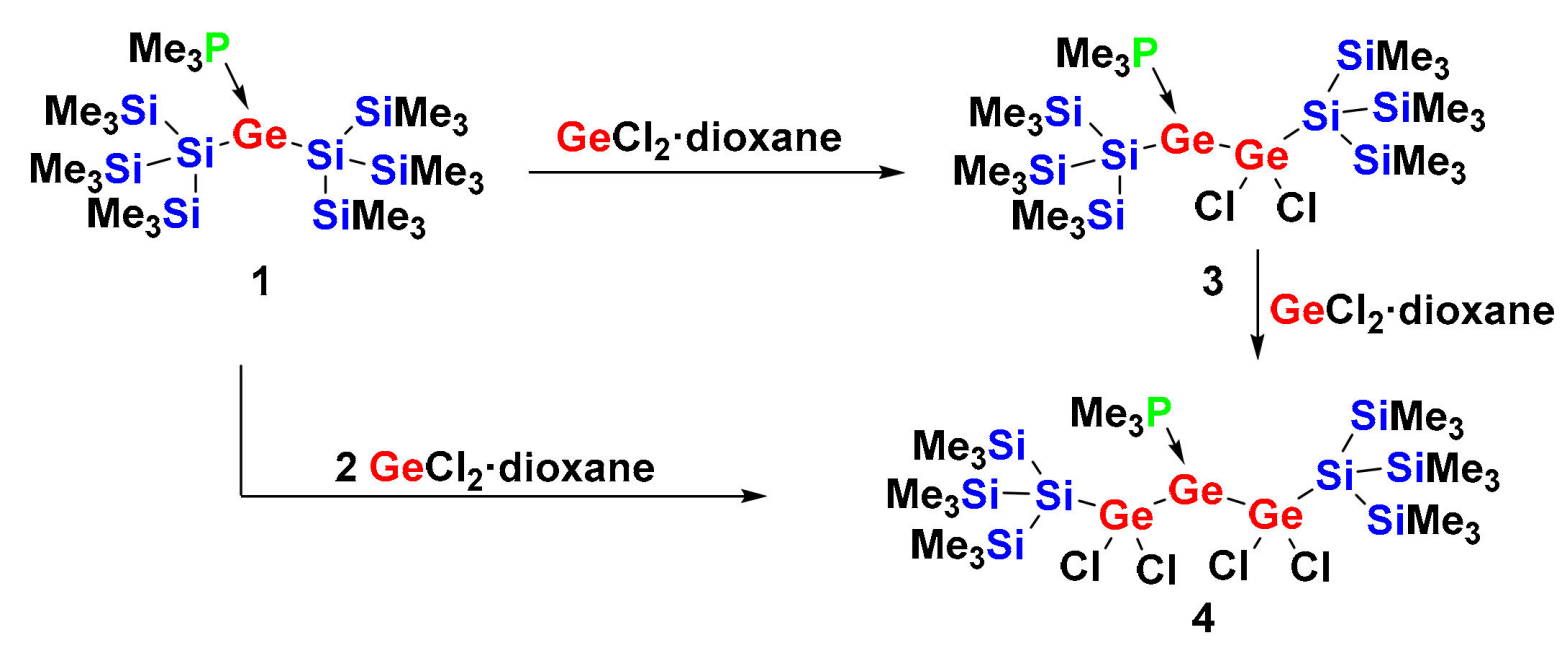
Single and double 1,1-migratory insertion reactions of GeCl_2_·dioxane into the Ge–Si bonds of disilylated germylene PMe_3_ adduct **1**.

**Figure 1:**
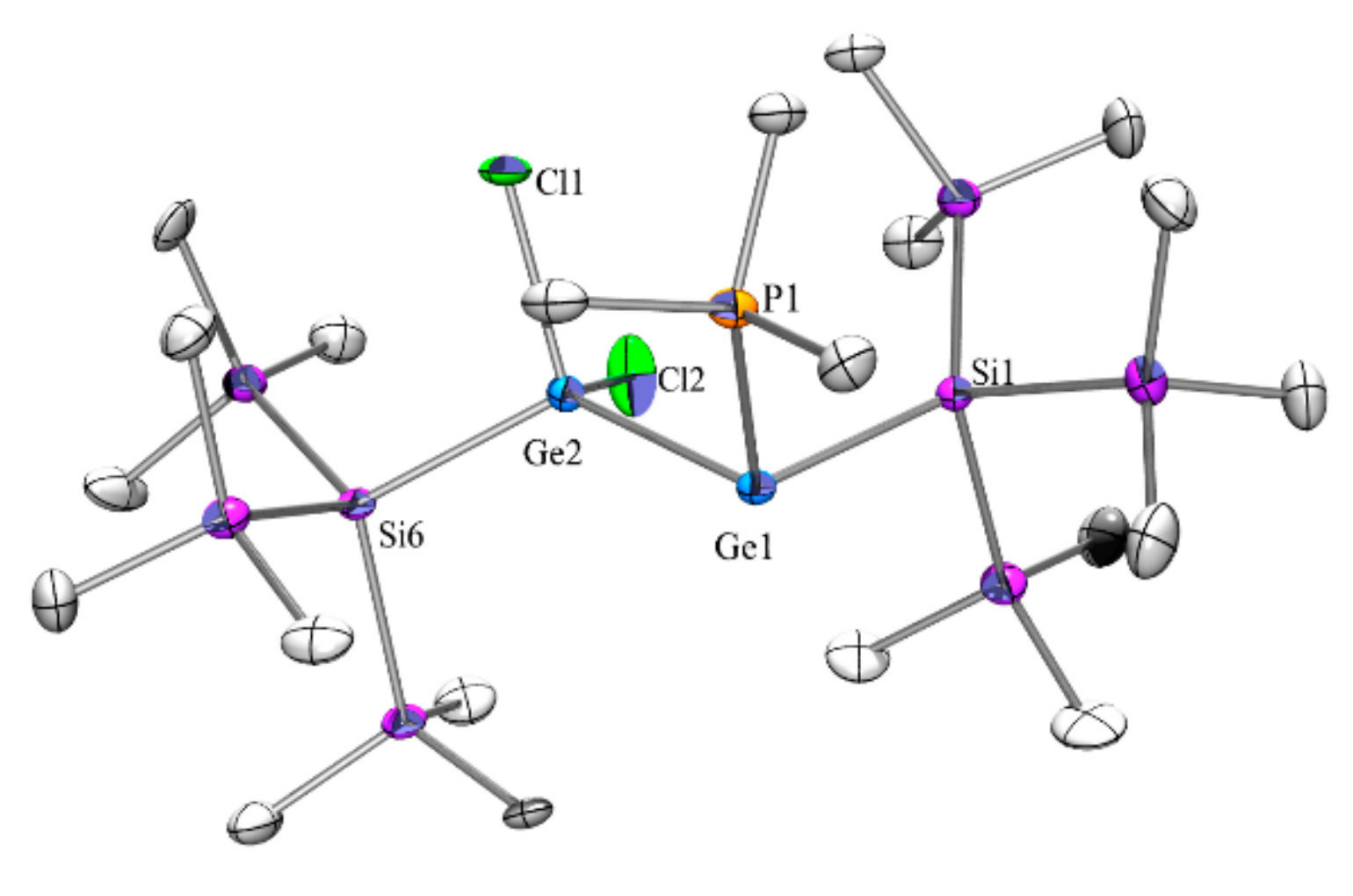
The molecular structure of **3** (only one of two independent molecules in the asymmetric unit is shown; the thermal ellipsoid plot is drawn at the 30% probability level). All hydrogen atoms omitted for clarity (bond length in Å, angles in deg). Ge(1)–P(1) 2.380(3), Ge(1)–Si(1) 2.489(3), Ge(1)–Ge(2) 2.5210(18), Ge(2)–Cl(2) 2.208(4), Ge(2)–Cl(1) 2.253(3), Ge(2)–Si(6) 2.428(3), Si(1)–Si(2) 2.361(4), Si(2)–C(1) 1.864(14), P(1)–C(11) 1.807(13), P(1)–Ge(1)-Si(1) 99.69(11), P(1)–Ge(1)–Ge(2) 93.80(9), Si(1)–Ge(1)-Ge(2) 105.64(9), Cl(2)–Ge(2)-Cl(1) 99.49(17), Cl(2–Ge(2)-Si(6) 102.67(14), Cl(1)–Ge(2)–Si(6) 101.21(12), Cl(2)–Ge(2)–Ge(1) 108.28(12), Cl(1)–Ge(2)-Ge(1) 116.19(10), Si(6)–Ge(2)–Ge(1) 125.38(10).

**Figure 2:**
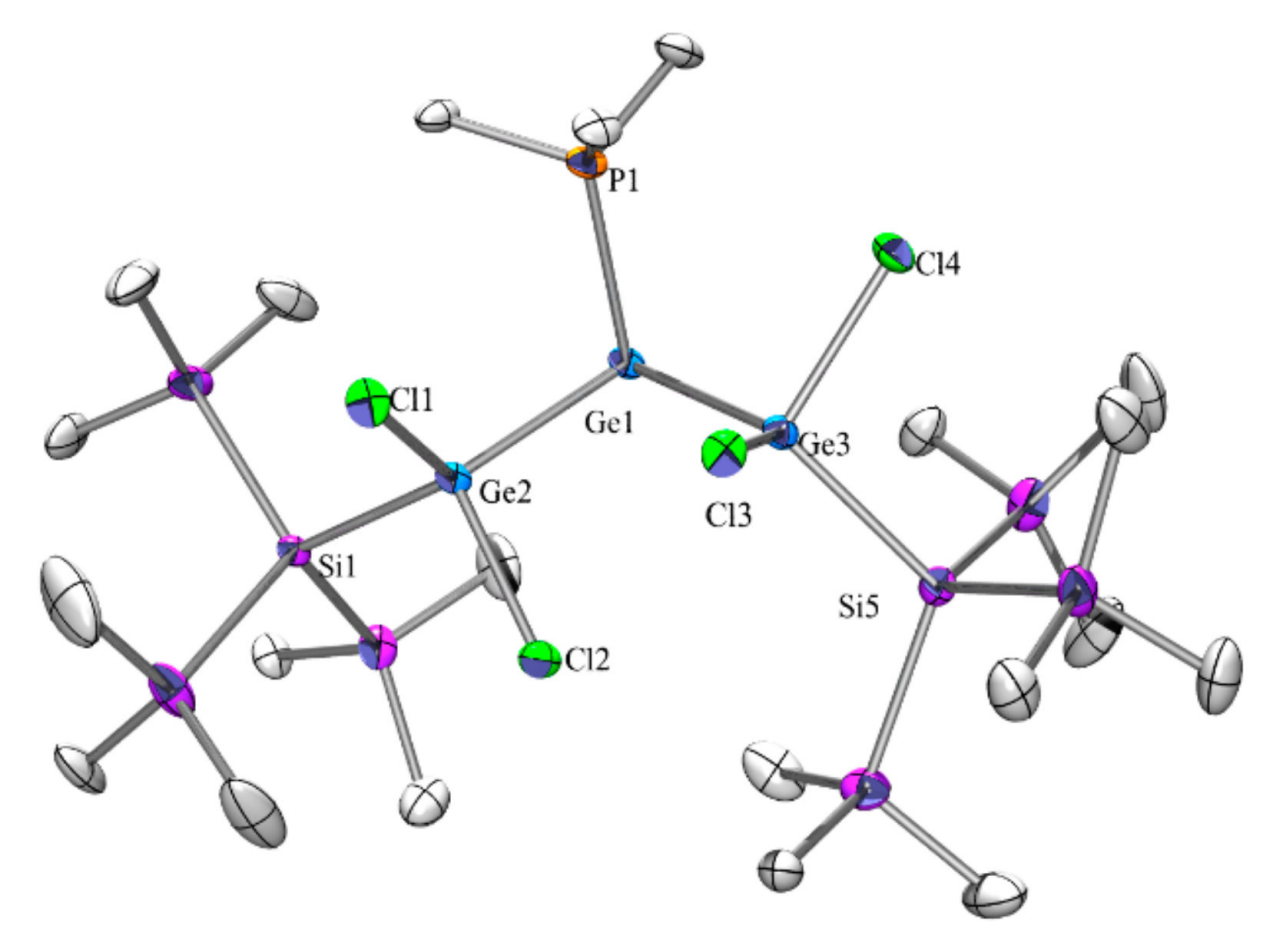
Molecular structure of **4** (thermal ellipsoid plot drawn at the 30% probability level; one only partially (ca. 75%) occupied pentane molecule was observed in the asymmetric unit). All hydrogen atoms omitted for clarity (bond lengths in Ả, angles in deg). Ge(1)–P(1) 2.3900(16), Ge(1)–Ge(2) 2.4980(9), Ge(1)–Ge(3) 2.5114(9), Ge(2)–Cl(1) 2.2177(15), Ge(2)–Cl(2) 2.2228(15), Ge(2)–Si(1) 2.3966(15), Ge(3)–Cl(3) 2.2149(15), Ge(3)–Cl(4) 2.2397(16), Ge(3)–Si(5) 2.4101(18), P(1)–C(20) 1.810(6), Si(1)–Si(2) 2.339(2), Si(2)–C(1) 1.860(8), P(1)–Ge(1)-Ge(2) 96.91(5), Ge(2)–Ge(1)-Ge(3) 97.31(3), Si(1)–Ge(2)–Ge(1) 126.78(4), Si(5)–Ge(3)-Ge(1) 128.04(5).

The reaction of **1** with one equivalent of GeCl_2_·dioxane resulted in the formation of compound **3** (Scheme 2, Figure 1), which can be considered a 1,1-migratory insertion product of GeCl_2_ into an Si–Ge bond of **1**. The further reaction of **3** with another equivalent of GeCl_2_·dioxane caused the selective 1,1-insertion of GeCl_2_ into the other Si–Ge bond of **3** to give the digermylgermylene adduct **4** (Figure 2).

Insertion reactions of GeCl_2_ into Ge-element bonds are not very common but have been reported on a few occasions. Most notable is the isomerization of Cl_3_GeGeCl_3_ to GeCl_4_ and GeCl_2_, followed by the four-fold insertion of GeCl_2_ into all Ge–Cl bonds of GeCl_4_ to give neopentagermane Ge(GeCl_3_)_4_ [19]. More recently, Rivard and co-workers described the related reaction of an NHC adduct of GeCl_2_ with GeCl_2_ to give branched oligomers [20]. The insertion of GeCl_2_·dioxane into the Ge–P bond of a germylene phosphane adduct was reported by Wesemann and co-workers [21], whereas Schnepf and co-workers observed insertion of GeCl_2_·dioxane into the S–C bonds of [(Me_3_Si)_3_CS]_2_Ge: [22].

In the solid state structure of **3** (Figure 1), the elongation of bond distances of divalent germanium to neighboring atoms (Ge(1)–Ge(2) 2.5210(18) Å, Ge(2)–Si(6) 2.428(3) Å) was not as pronounced as was observed before for related cyclic and acyclic compounds [5,23,24]. Consistently, the structure of **4** (Figure 2) displayed elongated Ge–Ge bonds of 2.4980(9) and 2.5114(9) Å.

**Scheme 3:**
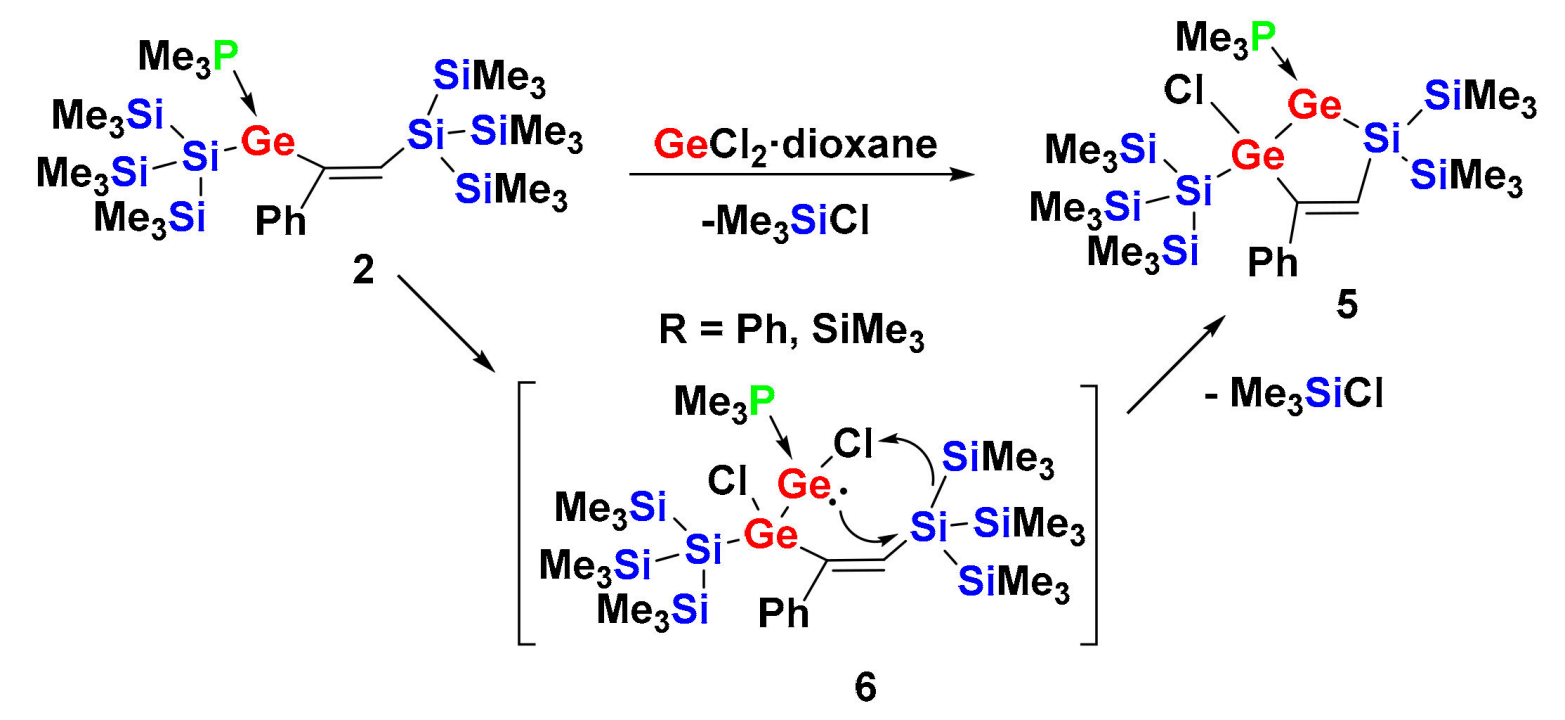
Reaction of vinylgermylene PMe_3_ adduct **2** with GeCl_2_·dioxane to a cyclic PMe_3_ germylene adduct by initial chlorogermylene adduct formation and subsequent Me_3_SiCl elimination.

**Figure 3:**
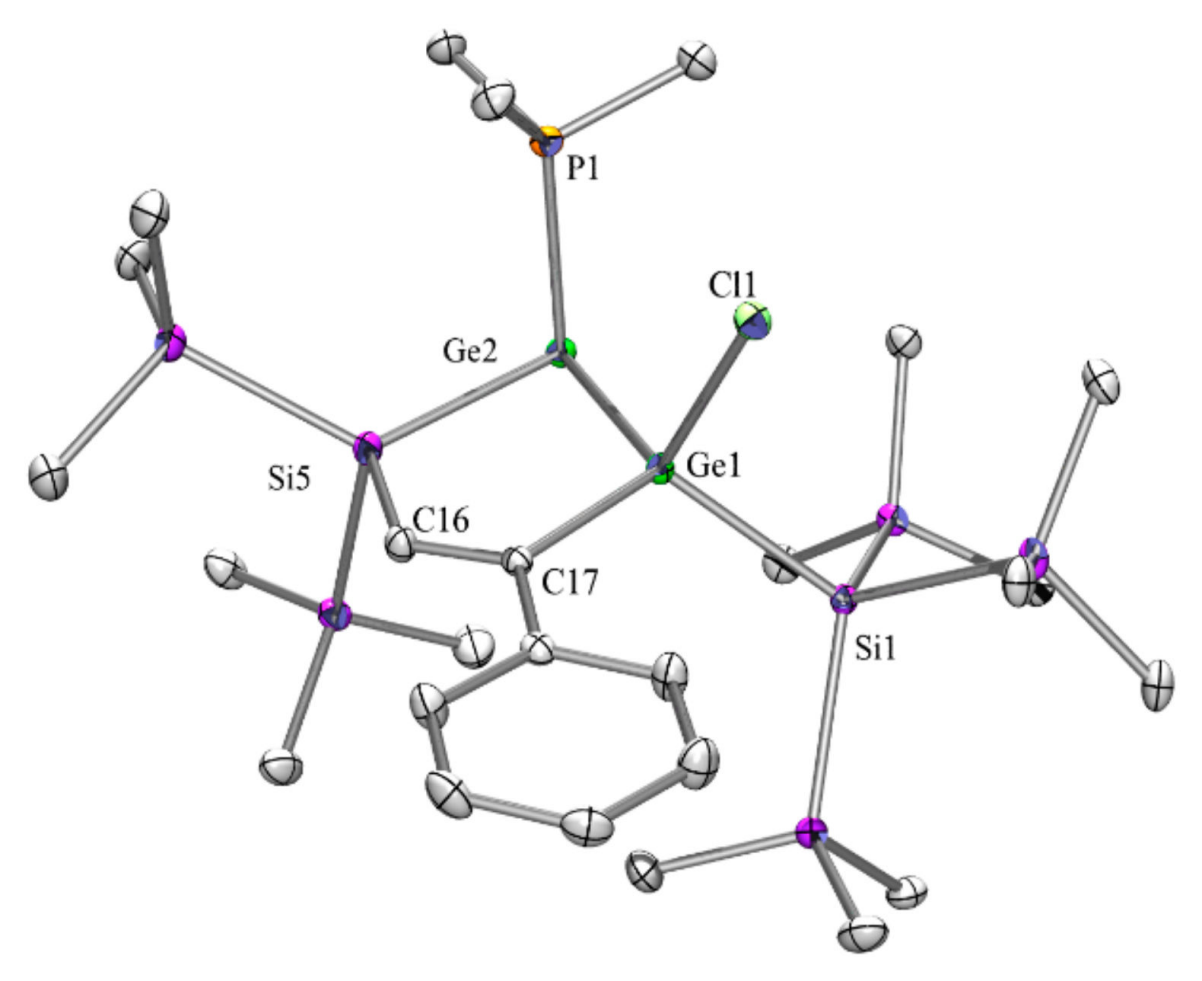
Molecular structure of **5** (thermal ellipsoid plot drawn at the 30% probability level). All hydrogen atoms omitted for clarity (bond lengths in Å, angles in deg). Ge(1)–C(17) 1.997(2), Ge(1)–Cl(1) 2.2725(8), Ge(1)–Si(1) 2.4285(8), Ge(1)–Ge(2) 2.5052(5), Ge(2)–P(1) 2.3610(9), Ge(2)–Si(5) 2.4409(9), P(1)–C(25) 1.814(3), Si(1)–Si(2) 2.3645(12), Si(2)–C(1) 1.879(3), Si(5)–C(16) 1.891(3), C(17)–Ge(1)–Cl(1) 103.05(8), C(17)–Ge(1)–Si(1) 118.48(8), C(17)–Ge(1)–Ge(2) 106.59(7), Si(1)–Ge(1)–Ge(2) 115.24(2), Si(5)–Ge(2)–Ge(1) 84.97(3).

In the ^29^Si NMR spectrum of **3**, the Ge(PMe_3_)Si(SiMe_3_)_3_ part (δ = −8.7 (d, ^3^*J*_Si-P_ = 11.4 Hz, GeSi(*Si*Me_3_)_3_) and −112.6 (d, ^2^*J*_Si-P_ = 16.1 Hz, *Si*(SiMe_3_)_3_) very much resembles the spectrum of **1**, while the GeCl_2_ unit attached to the second Si(SiMe_3_)_3_ group caused a deshielding effect of the chemical shift for the central silicon atom of the Si(SiMe_3_)_3_ unit to −76.7 ppm. The same was observed for the signal at −73.0 ppm in the ^29^Si NMR spectrum of **4**. With the phenylacetylene insertion product **2** readily available [5], we were interested in whether the 1,1-migratory insertion of GeCl_2_ into the remaining Ge–Si bond of **2** would occur with similar ease. The reaction of vinylgermylene PMe_3_ adduct **2** with GeCl_2_·dioxane indeed proceeded quantitatively, however, the obtained compound **5** (Figure 3, Scheme 3) was not the expected insertion product of the Ge–Si bond, but rather a cyclic germylene PMe_3_ adduct.

It is known that germylene phosphane adducts are in equilibrium with the free germylene [13]. For the formation of **5** we assumed that, in the first step, the respective free germylene of **2** inserted into a Ge–Cl bond of GeCl_2_, forming a germylchlorogermylene PMe_3_ adduct intermediate (**6**). The intramolecular cyclization of **6** can occur via the metathesis of a Ge–Cl with a Si–SiMe_3_ bond, releasing Me_3_SiCl with simultaneous Si–Ge bond formation (Scheme 3). In this mechanism, the eventual germylene involves not the original germanium atom but the one introduced by the addition of GeCl_2_.

In the solid state structure of **5,** the planar ring features of Ge–Ge (2.5052(5) Å) and Ge–P (2.3610(9) Å) bond distances, which are somewhat shorter than that of **3** and **4**, were the bond of the germylene atom to the Si(SiMe_3_)_2_ unit (2.4409(9) Å), which is longer than in **3** but still short of the 2.4816(8) Å observed for **2**. The Si–Ge–Ge angle is very acute (84.97(3)°), with the Ge–PMe_3_ interaction located almost orthogonal to the ring plane. The ^29^Si NMR spectrum features the signal for the germylene attached *Si*(SiMe_3_)_2_ unit at −50.1 ppm (d, ^2^*J*_Si-P_ = 15 Hz) and that for the C*Si*(SiMe_3_)_3_ at −100.7 (d, ^3^*J*_Si-P_ = 2 Hz).

Both NMR spectroscopic results and XRD data showed selective formation of only one isomer of **5**. It is reasonable to assume that the other possible isomer where PMe_3_ and Cl are on different sides of the ring plane is less stable. Since we have previously shown that the dissociation of PMe_3_ from silylated germylenes is a facile process, re-association of PMe_3_ would occur in a selective way to form the more thermodynamically stable product [13].

**Scheme 4:**
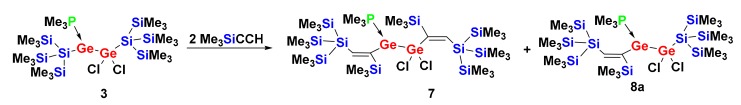
Reaction of the α-GeCl_2_ germylene PMe_3_ adduct **3** with Me_3_SiC≡CH to form another α-GeCl_2_ germylene PMe_3_ adduct (**7**) with two Me_3_SiC≡CH units inserted into Si–Ge bonds, which was isolated together with the intermediate product **8a**.

Compound **3** offered the opportunity to also study the reaction sequence of 1,1-insertion of GeCl_2_ followed by 1,2-insertion of an alkyne. In particular, the regioselectivity of the alkyne insertion reaction was of interest. As the germylene atom of adduct **3** has two weak bonds, the question arose whether the alkyne would insert into the Ge–Si bond or into the, presumably, weaker Ge–Ge bond. Much to our surprise, we observed two products. One was the double regioselective insertion of Me_3_SiC≡CH (**7**), not into the expected Ge–Si and Ge–Ge bonds adjacent to the germylene atom but into the two different Ge–Si bonds presented in **3** (Scheme 4). While the structure of compound **7** was unequivocally established by single crystal XRD analysis, the structure of the other compound **8a** was assigned by NMR spectroscopy as the product of a single alkyne insertion into the germylene–Si bond.

**Scheme 5:**
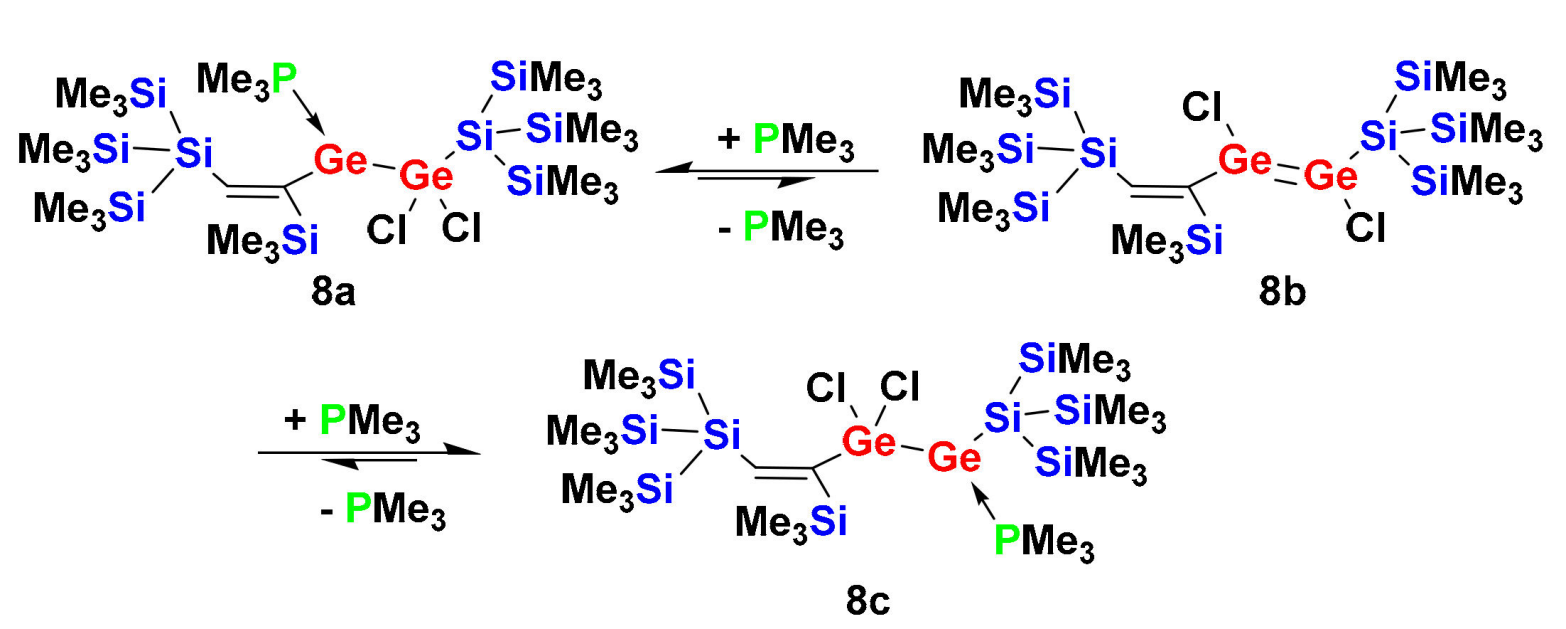
Mechanistic rationale for the formation of compound 7 involving the germylene-digermene rearrangement steps.

The outcome of the experiment was rationalized by assuming that the first step in the reaction occurred analogously to what is shown in Scheme 1. The 1,2-insertion of the alkyne into the (Me_3_Si)_3_Si–Ge bond gave the germylene adduct **8a** (Scheme 5). The initial formation of **8a** was consistent with NMR spectroscopic analysis of the reaction course. Dissociation of PMe_3_ can lead to the free germylene of **8a**, which can rearrange to 1,2-dichlorodigermene **8b** and, in a further rearrangement step, lead to isomer **8c**, where formally the two germanium atoms of **8a** and **8c** exchanged valency (Scheme 5). The insertion of another equivalent of Me_3_SiC≡CH into the (Me_3_Si)_3_Si–Ge bond of isomer **8c** led to **7**.

**Figure 4:**
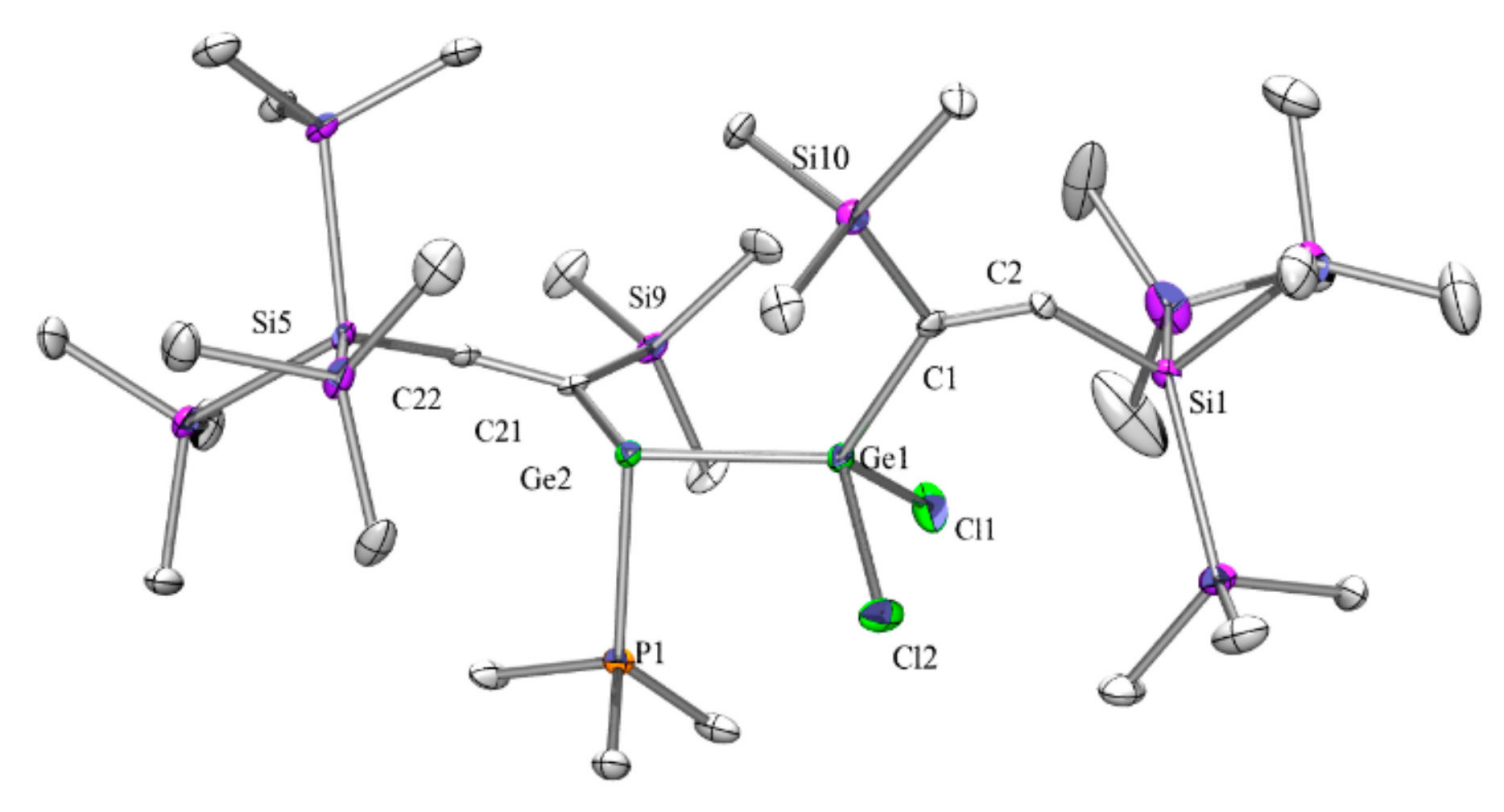
Molecular structure of **7** (thermal ellipsoid plot drawn at the 30% probability level). All hydrogen atoms omitted for clarity (bond lengths in Å, angles in deg). Ge(1)–C(1) 1.981(9), Ge(1)–Cl(1) 2.214(3), Ge(1)–Cl(2) 2.225(3), Ge(1)–Ge(2) 2.5178(14), Ge(2)–C(21) 2.016(8), Ge(2)–P(1) 2.431(2), P(1)–C(28) 1.815(9), Si(1)–C(2) 1.925(9), Si(1)–Si(2) 2.343(4), Si(5)–C(22) 1.872(9), Si(10)–C(1) 1.893(9), C(1)–C(2) 1.342(11), C(21)–C(22) 1.371(10), C(1)–Ge(1)–Ge(2) 120.6(3), Cl(1)–Ge(1)–Ge(2) 122.41(9), Cl(2)–Ge(1)–Ge(2) 101.26(9), C(21)–Ge(2)–P(1) 96.8(2), C(21)–Ge(2)–Ge(1) 110.0(3), P(1)–Ge(2)–Ge(1) 91.77(7).

The solid state structure of **7** (Figure 4) again revealed elongated distances to the divalent germylene atom of 2.5178(14) and 2.016(8) Å for the Ge–Ge and Ge–C bonds. It is interesting to note that, within the molecule, seven consecutively connected atoms (P–Ge–Ge–C=C–Si–Si) were almost coplanar, with a second plane defined by Ge–C=C–Si–S perpendicular to the first. The ^29^Si NMR spectroscopic analysis of the intermediate **8a** showed that the signal for the silicon atom attached to the GeCl_2_ unit at −78.6 ppm was close to the related signal of **3**, whereas the central silicon atom of the vinylated Si(SiMe_3_)_3_ group appeared at a typical chemical shift of −87.3 ppm. For the final product **7**, these signals were observed at −85.3 and −95.0 ppm. 

**Figure 5:**
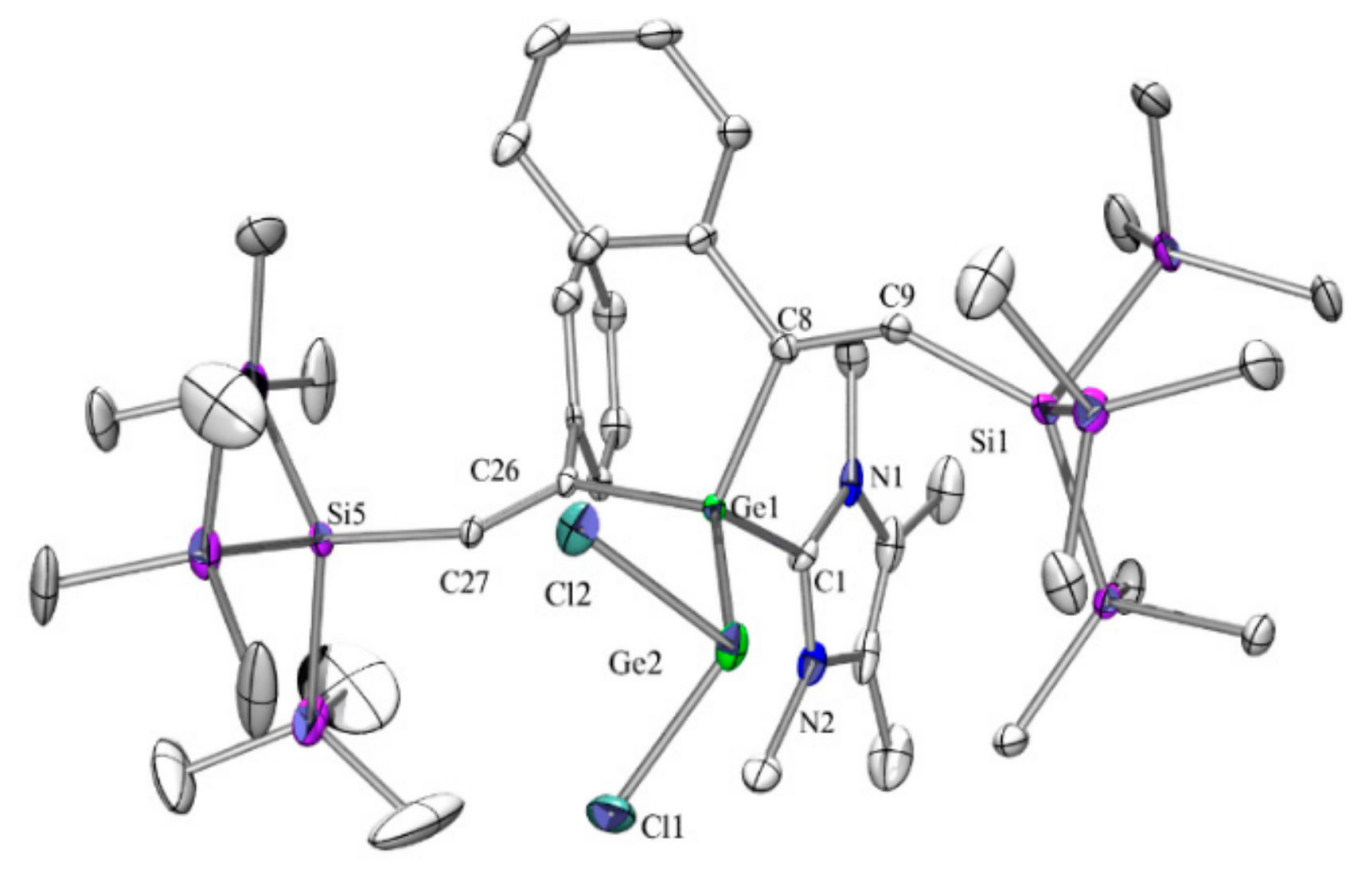
Molecular structure of **10** (thermal ellipsoid plot drawn at the 30% probability level). All hydrogen atoms and one molecule of THF in the asymmetric unit omitted for clarity (bond lengths in Å, angles in deg). Ge(1)–C(26) 1.995(4), Ge(1)–C(8) 1.999(4), Ge(1)–C(1) 2.006(4), Ge(1)–Ge(2) 2.5628(8), Ge(2)–Cl(2) 2.2997(14), N(1)–C(1) 1.354(6), Si(1)–C(9) 1.890(5), Si(1)–Si(2) 2.3539(19), Si(5)–C(27) 1.897(4), C(8)–C(9) 1.344(6), C(26)–C(27) 1.336(6), C(26)–Ge(1)–C(8) 111.56(17), C(26)–Ge(1)–C(1) 96.80(17), C(8)–Ge(1)–C(1) 105.12(18), C(26)–Ge(1)–Ge(2) 122.36(12), C(8)–Ge(1)–Ge(2) 101.24(13), C(1)–Ge(1)–Ge(2) 119.04(13), N(2)–C(1)–N(1) 105.9(4), C(8)–C(9)–Si(1) 143.6(4), C(26)–C(27)–Si(5) 132.7(3).

**Scheme 6:**
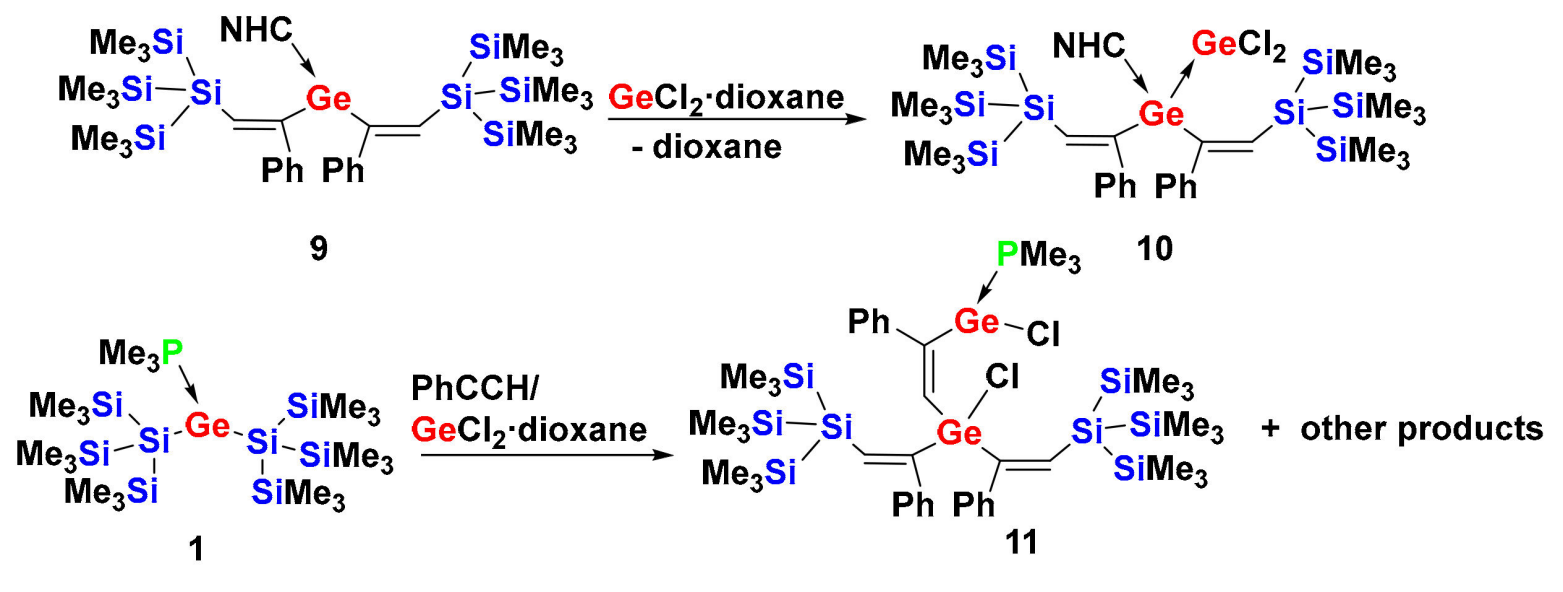
Top reaction: Reaction of divinyl germylene NHC adduct **9** with GeCl_2_·dioxane resulting in the formation of **10**, where the NHC adduct **9** acts as a Lewis base to GeCl_2_. Bottom reaction: Reaction of germylene **1** with three equivalents phenylacetylene and GeCl_2_·dioxane to give a trivinylated germane with a germylene PMe_3_ adduct moiety attached to one of the vinyl groups.

When an N-heterocyclic carbene (NHC) adduct of the double phenylacetylene insertion product **9** [13] was subjected to reaction with GeCl_2_·dioxane, the germylene lone pair served as a Lewis base to replace the dioxane of GeCl_2_ (**10**) (Scheme 6 top reaction, Figure 5). A similar reaction of Roesky’s germylene with GeCl_2_·dioxane has been reported [25].

The solid state structure of **10** (Figure 5) formally includes two divalent germanium atoms. Ge1 acts as a Lewis acid to accept the NHC as a base (Ge1–C1 = 2.006(4) Å). In addition Ge1 donates via its lone pair to the GeCl_2_ unit (Ge(1)–Ge(2) 2.5628(8) Å). Compared to the GeCl_2_ units in compounds **3**, **4** and **7**, the Ge–Cl distances of **10** were elongated to 2.30 Å, consistent with the divalent character of the germylene atom. As expected, the NMR spectra of **10** were fairly similar to that of **9**. The most pronounced difference was found for the ^13^C signal of the NHC carbene carbon atom, while for **9** a value of 179.9 ppm was observed, the respective signal for **10** can be detected at 150.6 ppm. Similar up-field shifts of the NHC carbene signal were observed previously for cases where the lone pair of a tetrylene NHC adduct acted as a base to a Lewis acid [26]. 

**Figure 6:**
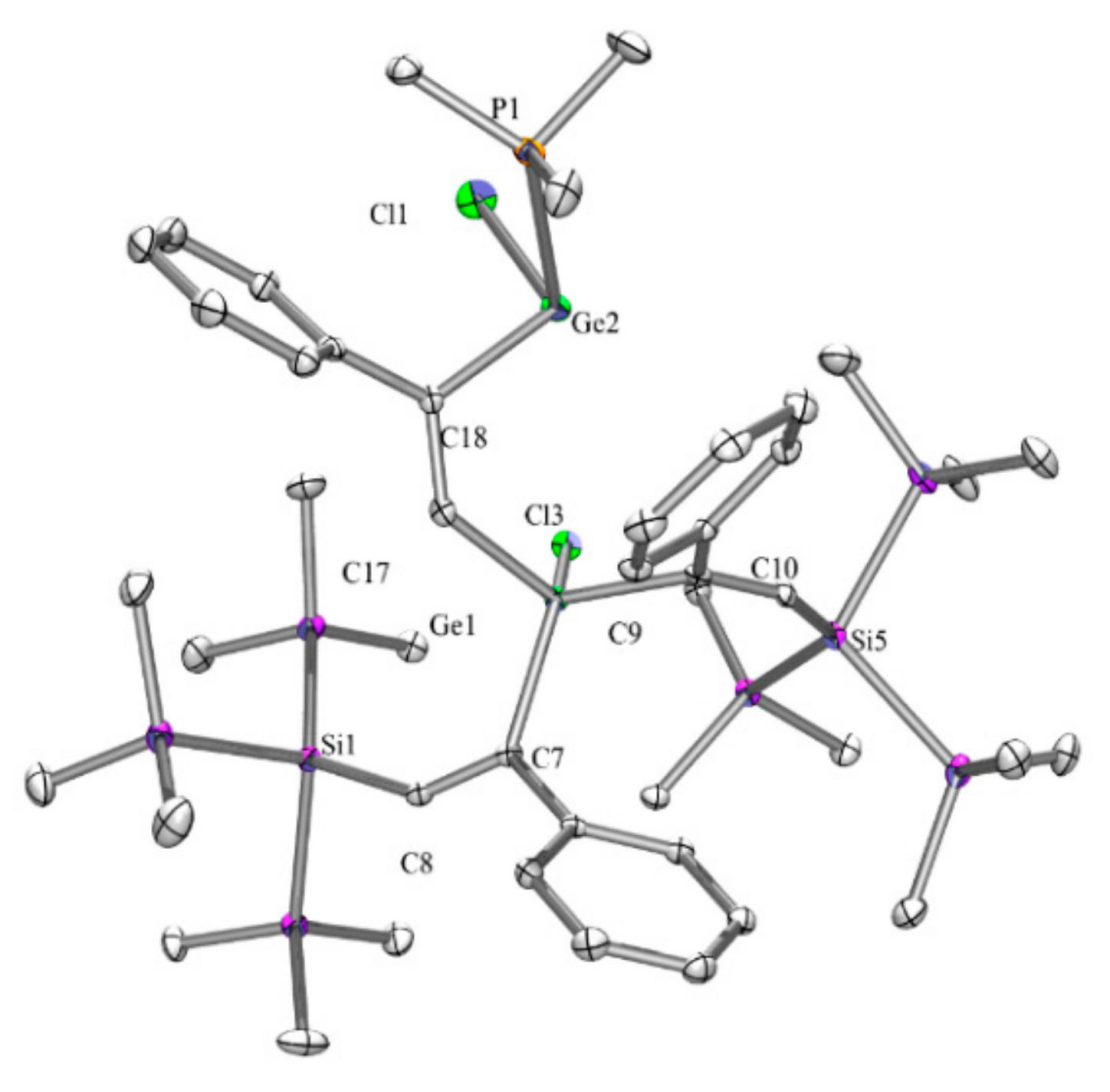
Molecular structure of **11** (thermal ellipsoid plot drawn at the 30% probability level). All hydrogen atoms are omitted for clarity (bond lengths in Å, angles in deg). Ge(1)–C(17) 1.942(3), Ge(1)–C(9) 1.961(3), Ge(1)–C(7) 1.963(3), Ge(1)–Cl(3) 2.1662(9), Ge(2)–C(18) 2.031(3), Ge(2)–Cl(1) 2.2962(11), Ge(2)–P(1) 2.4306(10), P(1)–C(43) 1.802(4), Si(1)–C(8) 1.905(3), Si(1)–Si(2) 2.3654(14), Si(5)–C(10) 1.897(3), C(7)–C(8) 1.340(4), C(9)–C(10) 1.329(4), C(17)–C(18) 1.327(4), C(17)–Ge(1)–C(9) 116.70(13), C(17)–Ge(1)–C(7) 104.41(13), C(9)–Ge(1)–C(7) 113.29(13), C(7)–C(8)–Si(1) 142.8(3), C(9)–C(10)–Si(5) 144.3(3).

In a related reaction, we reacted germylene adduct **1** with three equivalents phenylacetylene and one equivalent GeCl_2_·dioxane. Unfortunately, the reaction was not very selective but it was possible to select a crystal of PMe_3_ germylene adduct **11** under the microscope (Scheme 6, Figure 6). It seems reasonable that **11** was formed via the initial formation of the previously described divinylgermylene [5,13]. In a similar way, as was proposed for the formation of **5**, insertion of the divinylgermylene into a Ge–Cl bond of GeCl_2_·dioxane was supposed to give a chlorogermylgermylene. Phenylacetylene can now insert into the Ge–Ge bond of this new germylene, which eventually is isolated as its PMe_3_ adduct **11**. The germanium atom of the initial germylene then attached to three vinyl substituents. The third vinyl group, however, contained the phenyl group in the 2-position as its regioselectivity was then determined by the newly introduced germylene atom.

The crystal structure of **11** (Figure 6) features a tetravalent germanium atom with the three Ge–C distances covering a range between 1.94 and 1.96 Å and a Ge–Cl distance of 2.1662(9) Å. Again, the Ge–C bond to the divalent germanium atom Ge2 is longer (2.031(3) Å) and the same is true for the Ge–Cl bond (2.296(1) Å). 

## 3. Experimental Section

General Remarks: All reactions involving air-sensitive compounds were carried out under an atmosphere of dry nitrogen or argon using either Schlenk techniques or a glove box. All solvents were dried using a column based solvent purification system [27]. Chemicals were obtained from different suppliers and used without further purification. Adducts **1** [5,13], **2** [5,13,14], and **9 [13]** as well as reagents 1,3,4,5-tetramethylimidazol-2-ylidene (ImMe_4_) [28] and GeCl_2_·dioxane [29] were prepared according to previously published procedures. 

The ^1^H (300 MHz), ^13^C (75.4 MHz), ^31^P (121.4 MHz), and ^29^Si (59.3 MHz) NMR spectra were recorded on a Varian INOVA 300 spectrometer and are referenced to tetramethylsilane (TMS). If not otherwise noted, the solvent used was C_6_D_6_ and the samples were measured at rt. In the case of the reaction samples, a D_2_O capillary was used to provide an external lock frequency signal. To compensate for the low isotopic abundance of ^29^Si, the INEPT pulse sequence was used for the amplification of the signal [30,31].

Elementary analysis was carried out using a Heraeus VARIO ELEMENTAR. For a number of compounds, the obtained elemental analysis showed carbon values that were too low, which is a typical problem for these compounds likely caused by silicon carbide formation during the combustion process.

X-ray Structure Determination: For X-ray structure analyses, the crystals were mounted onto the tip of glass fibers. Data collection was performed with a BRUKER-AXS SMART APEX CCD diffractometer using graphite-monochromated Mo Kα radiation (0.71073 Å). The data were reduced to F^2^_o_ and corrected for absorption effects with SAINT [32] and SADABS [33,34], respectively. The structures were solved by direct methods and refined by full-matrix least-squares method (SHELXL97) [35]. If not noted otherwise all non-hydrogen atoms were refined with anisotropic displacement parameters and all hydrogen atoms were located in calculated positions to correspond to standard bond lengths and angles. Crystallographic data (excluding structure factors) for the structures of compounds **3**, **4**, **5**, **7**, **10**, and **11** reported in this paper were deposited with the Cambridge Crystallographic Data Center as supplementary publication number CCDC-969562 (**3**), 969565 (**4**), 1918810 (**5**), 1918811 (**7**), 1918808 (**10**), and 1918809 (**11**) (see Supplementary Materials). The data can be obtained free of charge at: http://www.ccdc.cam.ac.uk/products/csd/request/. The figures of solid state molecular structures were generated using Ortep-3 as implemented in WINGX [36] and rendered using POV-Ray 3.6 [37].

### 3.1. Dichloro[tris(trimethylsilyl)silyl]germyl[tris(trimethylsilyl)silyl]germylene·PMe_3_ Adduct (***3***) 

Method A: A solution of tris(trimethylsilyl)silyl potassium (0.31 mmol) in THF (2 mL) was slowly added dropwise to a vigorously stirred solution of GeCl_2_·dioxane (72 mg, 0.31 mmol) and trimethylphosphane (11 mg, 0.16 mmol) in THF (40 mL) at −30 °C. Stirring of the reaction mixture was continued for 1 h at rt. After the solvent was removed, the residue was extracted with pentane (3 × 5 mL). The orange extract was concentrated to 3 mL and stored at −35 °C. Yellow crystalline **3** (54 mg, 43%) was obtained. 

Method B: GeCl_2_·dioxane (194 mg, 0.84 mmol) in THF (2 mL) was added to a stirred solution of **1** (1.40 mmol) in THF (15 mL) at −30 °C. After stirring of the reaction mixture for 1 h at rt, further work-up proceeded analogous to Method A. Yellow crystalline **3** (376 mg, 56%) was obtained.

Mp = 116–119 °C. NMR: δ in ppm: ^1^H: 1.20 (d, 9H, ^2^*J*_H-P_ = 10.7 Hz, P*Me*_3_), 0.46 (s, 27H, Si*Me*_3_), 0.43 (s, 27H, Si*Me*_3_). ^13^C{^1^H}: 17.1 (d, ^1^*J*_C-P_ = 25.3 Hz, P*Me*_3_), 3.1 (Si*Me*_3_), 4.2 (Si*Me*_3_). ^29^Si{^1^H}: −8.2 (GeCl_2_Si(*Si*Me_3_)_3_), −8.7 (d, ^3^*J*_Si-P_ = 11.4 Hz, GeSi(*Si*Me_3_)_3_), −76.7 (Ge*Si*(SiMe_3_)_3_), −112.6 (d, ^2^*J*_Si-P_ = 16.1 Hz, *Si*(SiMe_3_)_3_). ^31^P{^1^H}: −15.2. Anal. calcd for C_21_H_63_Cl_2_Ge_2_PSi_8_ (787.55): C 32.03, H 8.06. Found: C 31.93, H 8.09. 

### 3.2. Bis{dichloro[tris(trimethylsilyl)silyl]germyl}germylene·PMe_3_ Adduct (***4***)

GeCl_2_·dioxane (173 mg, 0.75 mmol) in THF (2 mL) was added to a stirred solution of **1** (289 mg, 0.40 mmol) in THF (10 mL) at −30 °C. After 10 min the solvent was removed and the product was extracted with pentane (3 × 5 mL). The solution was concentrated to 5 mL and stored at −35 °C. Pale yellow crystalline **4** (118 mg, 34%) was obtained. Mp = 90–92 °C. NMR: δ in ppm: ^1^H: 1.27 (d, 9H, ^2^*J*_H-P_ = 11.7 Hz, P*Me*_3_), 0.45 (s, 54H, Si*Me*_3_). ^13^C{^1^H}: 29.9 (P*Me*_3_), 2.9 (Si*Me*_3_). ^29^Si{^1^H}: −8.0 (*Si*Me_3_), −73.0 (*Si*(SiMe_3_)_3_). ^31^P{^1^H}: −8.7. Anal. calcd for C_21_H_63_Cl_4_Ge_3_PSi_8_ (931.08): C 27.09, H 6.82. Found: C 26.80, H 6.69.

### 3.3. 1,2-Digerma-3-sila-1-chloro-1-tris(trimethylsilyl)silyl-3,3-bis(trimethylsilyl)-5-phenylcyclopent-4-ene-2-ylidene−PMe3 Adduct (***5***)

GeCl_2_·dioxane (23 mg, 0.1 mmol) was added to a stirring solution of **2** (75 mg, 0.1 mmol) in THF (2 mL). After 2 h the solvent was removed and the residue was extracted with pentane (3 × 5 mL). The solution was concentrated to 0.5 mL and stored at −35 °C. Pale yellow crystalline **5** (57 mg, 74%) was obtained. Mp = 122–124 °C. NMR: δ in ppm: ^1^H: 7.90 (d, *J* = 7.7 Hz, 2H, *o-H*), 7.15 (d, *J* = 7.4 Hz, 2H, *m-H*), 7.08 (s, 1H, CH), 6.99 (t, *J* = 7.4 Hz, 1H, *p-H*), 1.12 (d, ^2^*J*_H-P_ = 10.7 Hz, P*Me*_3_), 0.34 (s, 9 H, Si*Me*_3_), 0.31 (s, 27 H, Si*Me*_3_), 0.15 (s, 9 H, Si*Me*_3_). ^13^C{^1^H}: 171.3, 147.4, 146.6, 128.2, 126.7, 17.0 (d, ^1^*J*_C-P_ = 26 Hz, P*Me*_3_), 3.0 (Si*Me*_3_), 1.9 (Si*Me*_3_), 1.2 (Si*Me*_3_). ^29^Si{^1^H}: −8.7 (*Si*Me_3_), −11.1 (d, ^3^*J*_Si-P_ = 13 Hz, *Si*Me_3_), −11.7 (d, ^3^*J*_Si-P_ = 17 Hz, *Si*Me_3_), −50.1 (d, ^2^*J*_Si-P_ = 15 Hz, Ge*Si*(SiMe_3_)_3_, −100.7 (d, ^3^*J*_Si-P_ = 2 Hz, *Si*(SiMe_3_)_2_). ^31^P{^1^H}: −13.9. Anal. calcd for C_26_H_60_Ge_2_Si_7_PCl (781.04): C 39.98, H 7.74. Found: C 39.74, H 7.70.

### 3.4. Adducts ***7*** and ***8a***

A solution of **3** (50 mg, 0.064 mmol) and trimethylsilylacetylene (10 mg, 0.11 mmol) in in THF (2 mL) was stirred at ambient temperature. After 2 h, the ^29^Si NMR suggested formation of the new product **8a** but still some **3** was present. After a further 12 h, all starting material was consumed and another new product was formed. When no further progress was detected, the solvent was removed with a vacuum and the residue dissolved in pentane (2mL). Light yellow crystals (30 mg) containing a mixture of **7** and **8a** formed after 12 h at −35 °C. Under the microscope, a suitable crystal of **7** was selected for single crystal XRD analysis. NMR: δ in ppm: Compound **8a** (THF/D_2_O-capillary): ^1^H: 7.67 (d, *J* = 2.5 Hz, 1H, C=C*H*), 1.58 (d, ^2^*J*_H-P_ = 9.7 Hz, P*Me*_3_), 0.39 (s, 9 H, Si*Me*_3_), 0.36 (s, 27 H, Si*Me*_3_), 0.27 (s, 9 H, Si*Me*_3_). ^29^Si{^1^H}: −8.2 (C*Si*Me_3_), −10.9 Si(*Si*Me_3_), −11.5 Si(*Si*Me_3_), −78.6 (GeCl_2_*Si*(SiMe_3_)_3_, −87.3 (C*Si*(SiMe_3_)_2_). ^31^P{1H}: −29.8. 

Compound **7** (C_6_D_6_): ^1^H: 8.47 (s, 1H, C=C*H*), 8.02 (s, 1H, C=C*H*), 0.79 (d, ^2^*J*_H-P_ = 7.5 Hz, P*Me*_3_), 0.34 (s, 27 H, Si(Si*Me*_3_)_3_), 0.32 (s, 27 H, Si(Si*Me*_3_)_3_), 0.26 (s, 9 H, Si*Me*_3_). 0.18 (s, 9 H, Si*Me*_3_). ^29^Si{^1^H}: −7.2 (C*Si*Me_3_), −8.2 (C*Si*Me_3_), −11.0 (Si(*Si*Me_3_)), −11.9 (Si(*Si*Me_3_)), −85.3 (*Si*(SiMe_3_)_3_, −95.0 (*Si*(SiMe_3_)_2_). ^31^P{^1^H}: −15.7.

### 3.5. Adduct ***10***

First, **9** (40 mg, 0.045 mmol) and GeCl_2_·dioxane (10 mg, 0.045 mmol) were stirred in THF (1 mL) at rt for 48 h. Next, pentane (1 mL) was added. After storage at −35 °C yellow crystalline **10** (22 mg, 47%) was obtained. Mp = 121–122 °C. NMR: δ in ppm: ^1^H: 7.12 (m, 2H), 6.99 (m, 4H), 6.61 (br, 4H), 6.28 (s, 1H), 3.33 (s, 6H, N–*Me*), 2.10 (s, 6H, C–*Me*), 0.20 (s, 54H, Si*Me*_3_). ^13^C{^1^H}: 150.6, 150.4, 147.0, 145.2, 130.8, 128.7, 127.5, 126.8, 34.0 (N–*Me*), 9.5 (C–*Me*), 1.5 (Si*Me*_3_). ^29^Si{^1^H}: −12.4 (*Si*Me_3_), −84.3 (Si_q_). Anal. calcd for C_41_H_78_Cl_2_Ge_2_N_2_Si_8_ (1039.94): C 47.35, H 7.56, N 2.69. Found: C 47.23, H 7.54, N 2.67.

### 3.6. Adduct ***11***

Phenylacetylene (87 mg, 0.85 mmol) was added to a stirred solution of **1** (0.27 mmol) in THF (5 mL) and after 1 h GeCl_2_·dioxane (86 mg, 0.37 mmol) was added. The completeness of conversion was determined by ^29^Si NMR after 24 h. The solvent was removed and the residue was extracted with toluene (5 × 5 mL). The solvent was removed to yield a brown solid (229 mg). Crystallization from toluene gave pale yellow crystals. ^29^Si NMR analysis indicated formation of at least four different compounds of which **11** is likely the major component. NMR: δ in ppm: ^29^Si{^1^H}: −11.5 (*Si*Me_3_), −84.9 (Si_q_). The selection of a well-shaped crystal from the batch under the microscope allowed the single crystal XRD analysis of **11**.

## 4. Conclusions

Here, we reported a few examples of a main group of analogs of what would be called 1,2-migratory insertions in transition metal chemistry. Mono-substituted alkynes were found to insert into the Si–Ge bonds of disilylated germylene PMe_3_ adducts [5,13,14]. The current account extends this chemistry to main group analogs of 1,1-migratory insertions. Reactions of one or two equivalents of GeCl_2_·dioxane with the PMe_3_ adduct of [(Me_3_Si)_3_Si]_2_Ge: gave either the mono- or diinsertion product of GeCl_2_ insertion into the Ge–Si bonds. Only a few examples of insertion reactions of GeCl_2_ into weak E–E bonds of group 14 oligomers are known [19,20,21,22], and the current chemistry seems to provide an interesting pathway to precursors for unsaturated group 14 cluster molecules.

How subtle changes in the starting material can change the reaction course profoundly became evident when we subjected another silylated germylene to reaction with GeCl_2_·dioxane. This time the alkyne mono-insertion product (Me_3_Si)_3_SiGe:(Ph)C=CHSi(SiMe_3_)_3_ served as the precursor and, instead of the expected GeCl_2_ insertion into the Si–Ge bond, we observed the substrate acting as germylene inserting into a Ge–Cl bond. The resulting chlorogermylchlorogermylene reacted further by trimethylsilyl chloride elimination to a cyclic germylene adduct.

In an attempt to reverse the order of 1,1 and 1,2-insertion, we next reacted the GeCl_2_ mono-insertion product (Me_3_Si)_3_SiGe:GeCl_2_Si(SiMe_3_)_3_ with Me_3_SiC≡CH. An unexpected mixture of alkyne mono- and diinsertion products was obtained, where the first alkyne insertion proceeded into the Si–Ge bond of the germylene but the second insertion did not occur into the Ge–Ge bond as expected but into the Ge–Si bond of the second Si(SiMe_3_)_3_ unit. For this to happen, the involved germanium atoms needed to swap oxidation states, meaning that the two Cl atoms of the GeCl_2_ would migrate to the divalent Ge-atom. We assumed that this rearrangement proceeds via known 1,2-shifts of chlorides involving germylene-digermene-germylene interconversion.

As expected, in the eventual reaction of a divinylgermylene with GeCl_2_·dioxane no germylene insertion chemistry was observed. In this case only the exchange of the dioxane ligand of GeCl_2_ against the divinylgermylene base could be detected. A final one-pot reaction of [(Me_3_Si)_3_Si]_2_Ge:·PMe_3_ with one equivalent GeCl_2_·dioxane and three equivalents of phenylacetylene gave a number of products with an interesting trivinylated chlorogermane, where a chlorogermylene is attached to one of the vinyl units. We suppose that this compound forms by initial divinylgermylene formation, followed by insertion of this germylene into a Ge–Cl bond of GeCl_2_. The thus formed germylene with a germyl and a chloride substituent can insert a third alkyne into the Ge–Ge bond. Since the newly introduced Ge atom now acts as the germylene the regiochemistry of the last inserted vinyl unit is reversed with respect to the central germanium atom.

We think that the demonstrated chemistry bears the potential become a useful method in the chemistry of heavy group 14 compounds. The facile introduction of GeCl_2_ into molecules that already contain a certain degree of unsaturation opens an entry into precursor chemistry of interesting oligogermanium cluster chemistry.

## Supplementary Materials

The following are available online, Tabulated crystallographic data and ORTEP plots for compounds **3**, **4**, **5**, **7**, **10**, and **11** and ^29^Si and ^31^P NMR spectra of compounds **3**, **4**, **5**, **7**/**8a**, and **10**.
