# Supplementary material for: 1,2- and 1,1-Migratory Insertion Reactions of Silylated Germylene Adducts
Source: Molecules. 2020 Feb 6;25(3):686. doi: 10.3390/molecules25030686 (PMC7037258; doi:10.3390/molecules25030686)
Supplement: Supplementary file 1 [file molecules-25-00686-s001.pdf]

Supporting Info for

1,2- and 1,1-Migratory Insertion Reaction of Silylated Germylene Adducts

Małgorzata Walewska, Judith Baumgartner, Christoph Marschner

Institut für Anorganische Chemie, Technische Universität Graz, Stremayrgasse 9, 8010 Graz, Austria

Table 1. Crystallographic data for compounds **3**, **4**, **5**, **7**, **10**, and **11**.

	**3**	**4**	**5**	**7**	**10**	**11**
Empirical formula	$Ge_4P_2Cl_4C_{42}H_{126}Si_{16}$	$Ge_3PCl_4C_{25}H_{74}Si_8$	$Ge_2PClC_{26}H_{60}Si_7$	$Ge_2PCl_2C_{31}H_{83}Si_{10}$	$Ge_2ON_2Cl_2C_{45}H_{86}Si_8$	$Ge_2PCl_2C_{45}H_{81}Si_8$
M_w	1574.97	990.35	780.97	983.92	1111.96	1093.87
Temperature [K]	150(2)	100(2)	100(2)	100(2)	100(2)	100(2)
Size [mm]	0.25×0.22×0.08	0.35×0.22×0.16	0.21×0.18×0.13	0.22×0.08×0.06	0.35×0.22×0.16	0.48×0.10×0.10
Crystal system	triclinic	orthorhombic	orthorhombic	monoclinic	monoclinic	orthorhombic
Space group	P-1	P2(1)2(1)2(1)	Pbca	P2(1)/c	P2(1)/n	Pccn
a [Å]	14.280(3)	10.694(2)	20.615(5)	12.424(3)	20.953(4)	26.870(5)
b [Å]	15.683(3)	16.314(3)	12.857(3)	27.226(6)	11.089(2)	36.690(7)
c [Å]	19.427(4)	30.352(6)	31.315(7)	18.602(3)	26.753(5)	12.656(3)
α [°]	90.23(3)	90	90	90	90	90
β [°]	103.57(3)	90	90	117.562	98.04(3)	90
γ [°]	92.27(3)	90	90	90	90	90
V [$Å^3$]	4225(2)	5295(2)	8300(3)	5578(2)	6155(2)	12477(4)
Z	2	4	8	4	4	8
ρ_{calc} [gcm^{-3}]	1.238	1.242	1.250	1.172	1.200	1.165
Absorption coefficient [mm^{-1}]	1.826	2.121	1.769	1.437	1.250	1.255
F(000)	1656	2061	3280	2088	2352	4608
θ range	1.08<θ<25.00	1.34<θ<26.39	1.63<θ<26.37	1.44<θ<26.27	1.54<θ<26.36	0.94<θ<26.37
Reflections collected/unique	29917/14683	42073/10817	52700/8486	14654/10304	48115/12539	74307/12615
Completeness to θ [%]	98.6	99.8	100	96.3	99.7	98.9
Data/restraints/parameters	14683/0/655	10817/0/415	8486/0/352	10304/6/473	12539/0/563	12615/0/544
Goodness of fit on F^2	1.05	1.02	1.03	0.89	1.15	1.04
Final R indices [I>2σ(I)]	R1=0.106<br>*w*R2=0.260	R1=0.052<br>*w*R2=0.109	R1=0.039<br>*w*R2=0.085	R1=0.090<br>*w*R2=0.139	R1=0.072<br>*w*R2=0.154	R1=0.049<br>*w*R2=0.109
R indices (all data)	R1=0.152<br>*w*R2=0.278	R1=0.065<br>*w*R2=0.114	R1=0.052<br>*w*R2=0.090	R1=0.180<br>*w*R2=0.190	R1=0.086<br>*w*R2=0.162	R1=0.068<br>*w*R2=0.116
Largest diff. Peak/hole [e^-/ $Å^3$]	1.72/−1.20	0.98/−0.41	0.91/−0.34	1.15/−0.55	1.23/−0.75	1.96/−0.58

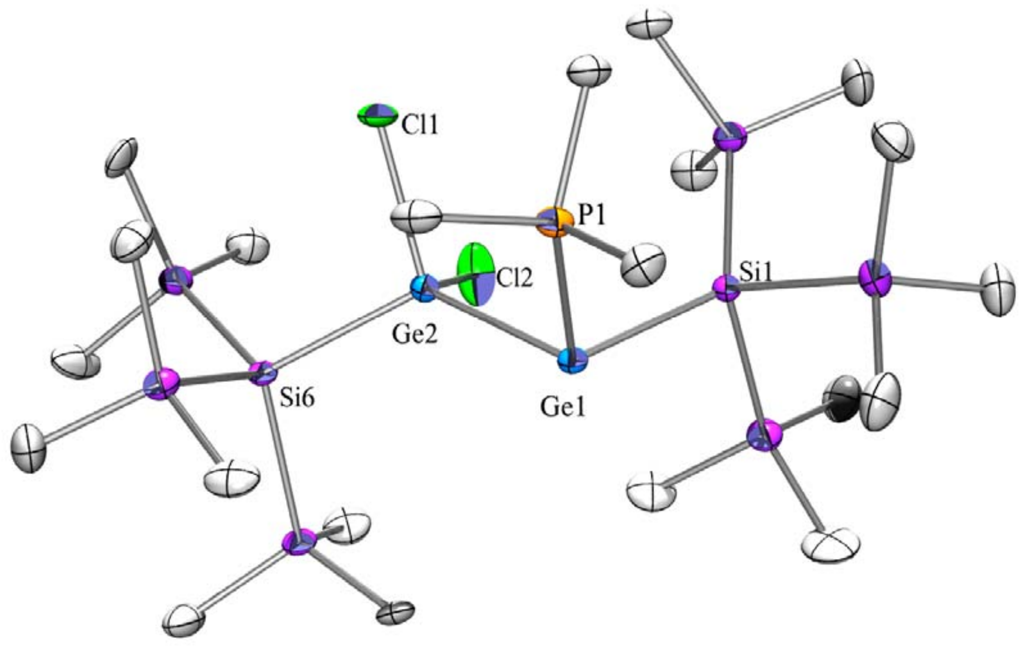

Figure 1. Molecular structure of **3** (thermal ellipsoid plot drawn at the 30% probability level). All hydrogen atoms are omitted for clarity (bond lengths in Å, angles in deg, two independent molecules in the asymmetric unit). Ge(1)-P(1) 2.380(3), Ge(1)-Si(1) 2.489(3), Ge(1)-Ge(2) 2.5210(18), Ge(2)-Cl(2) 2.208(4), Ge(2)-Cl(1) 2.253(3), Ge(2)-Si(6) 2.428(3), Si(1)-Si(2) 2.361(4), Si(2)-C(1) 1.864(14), P(1)-C(11) 1.807(13), P(1)-Ge(1)-Si(1) 99.69(11), P(1)-Ge(1)-Ge(2) 93.80(9), Si(1)-Ge(1)-Ge(2) 105.64(9), Cl(2)-Ge(2)-Cl(1) 99.49(17), Cl(2)-Ge(2)-Si(6) 102.67(14), Cl(1)-Ge(2)-Si(6) 101.21(12), Cl(2)-Ge(2)-Ge(1) 108.28(12), Cl(1)-Ge(2)-Ge(1) 116.19(10), Si(6)-Ge(2)-Ge(1) 125.38(10).

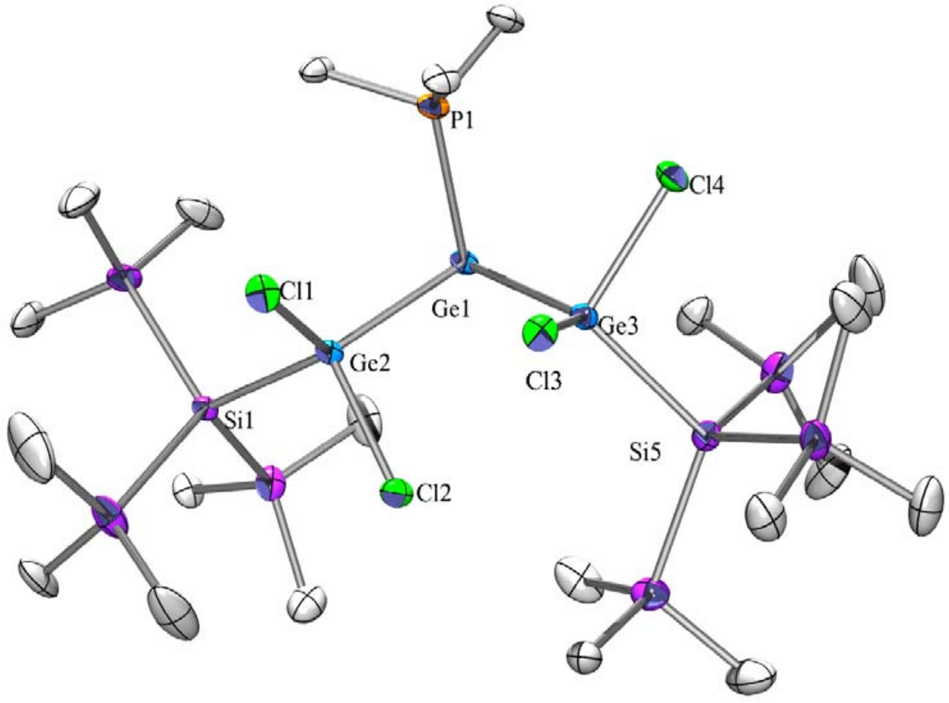

Figure 2. Molecular structure of **4** (thermal ellipsoid plot drawn at the 30% probability level, one only partially (ca 75%) occupied pentane molecule was observed in the asymmetric unit). All hydrogen atoms are omitted for clarity (bond lengths in Å, angles in deg). Ge(1)-P(1) 2.3900(16), Ge(1)-Ge(2) 2.4980(9), Ge(1)-Ge(3) 2.5114(9), Ge(2)-Cl(1) 2.2177(15), Ge(2)-Cl(2) 2.2228(15), Ge(2)-Si(1) 2.3966(15), Ge(3)-Cl(3) 2.2149(15), Ge(3)-Cl(4) 2.2397(16), Ge(3)-Si(5) 2.4101(18), P(1)-C(20) 1.810(6), Si(1)-Si(2) 2.339(2), Si(2)-C(1) 1.860(8), P(1)-Ge(1)-Ge(2) 96.91(5), Ge(2)-Ge(1)-Ge(3) 97.31(3), Si(1)-Ge(2)-Ge(1) 126.78(4), Si(5)-Ge(3)-Ge(1) 128.04(5).

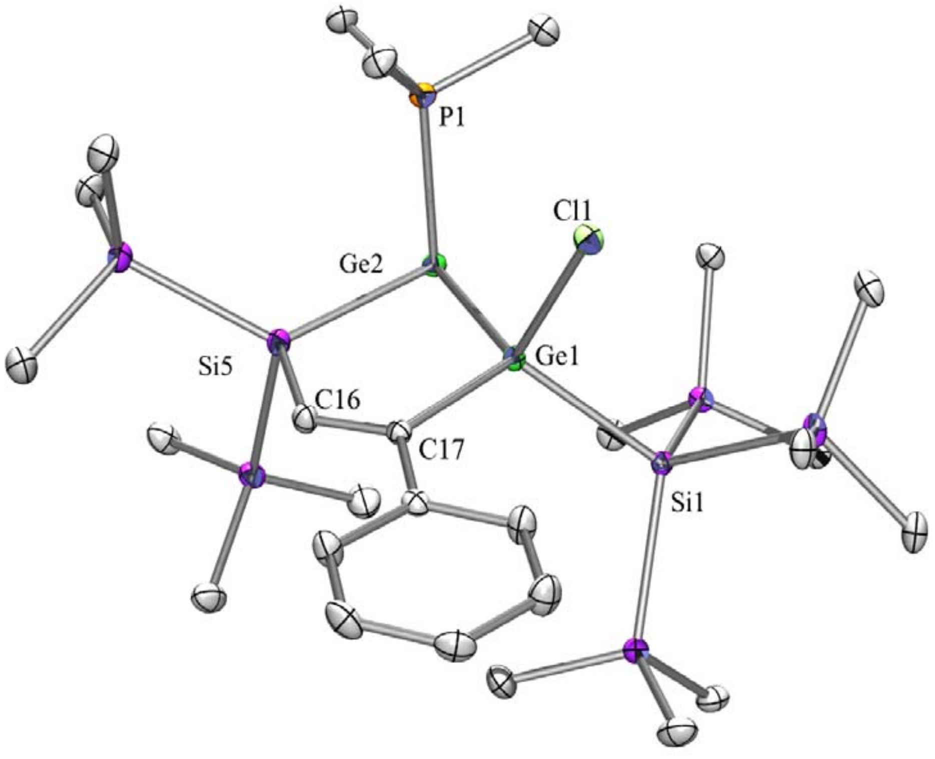

Figure 3. Molecular structure of **5** (thermal ellipsoid plot drawn at the 30% probability level). All hydrogen atoms are omitted for clarity (bond lengths in Å, angles in deg). Ge(1)-C(17) 1.997(2), Ge(1)-Cl(1) 2.2725(8), Ge(1)-Si(1) 2.4285(8), Ge(1)-Ge(2) 2.5052(5), Ge(2)-P(1) 2.3610(9), Ge(2)-Si(5) 2.4409(9), P(1)-C(25) 1.814(3), Si(1)-Si(2) 2.3645(12), Si(2)-C(1) 1.879(3), Si(5)-C(16) 1.891(3), C(17)-Ge(1)-Cl(1) 103.05(8), C(17)-Ge(1)-Si(1) 118.48(8), C(17)-Ge(1)-Ge(2) 106.59(7), Si(1)-Ge(1)-Ge(2) 115.24(2), Si(5)-Ge(2)-Ge(1) 84.97(3).

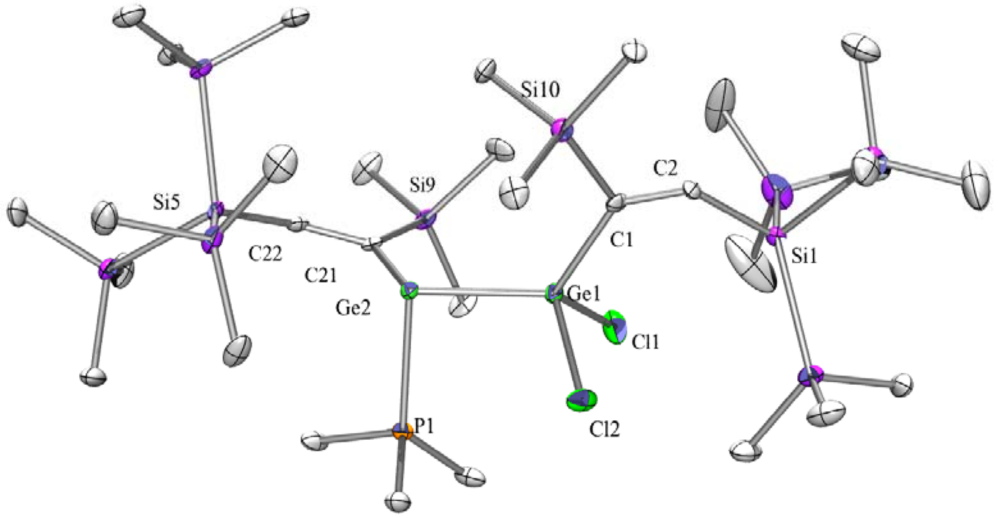

Figure 4. Molecular structure of **7** (thermal ellipsoid plot drawn at the 30% probability level). All hydrogen atoms are omitted for clarity (bond lengths in Å, angles in deg). Ge(1)-C(1) 1.981(9), Ge(1)-Cl(1) 2.214(3), Ge(1)-Cl(2) 2.225(3), Ge(1)-Ge(2) 2.5178(14), Ge(2)-C(21) 2.016(8), Ge(2)-P(1) 2.431(2), P(1)-C(28) 1.815(9), Si(1)-C(2) 1.925(9), Si(1)-Si(2) 2.343(4), Si(5)-C(22) 1.872(9), Si(10)-C(1) 1.893(9), C(1)-C(2) 1.342(11), C(21)-C(22) 1.371(10), C(1)-Ge(1)-Ge(2) 120.6(3), Cl(1)-Ge(1)-Ge(2) 122.41(9), Cl(2)-Ge(1)-Ge(2) 101.26(9), C(21)-Ge(2)-P(1) 96.8(2), C(21)-Ge(2)-Ge(1) 110.0(3), P(1)-Ge(2)-Ge(1) 91.77(7).

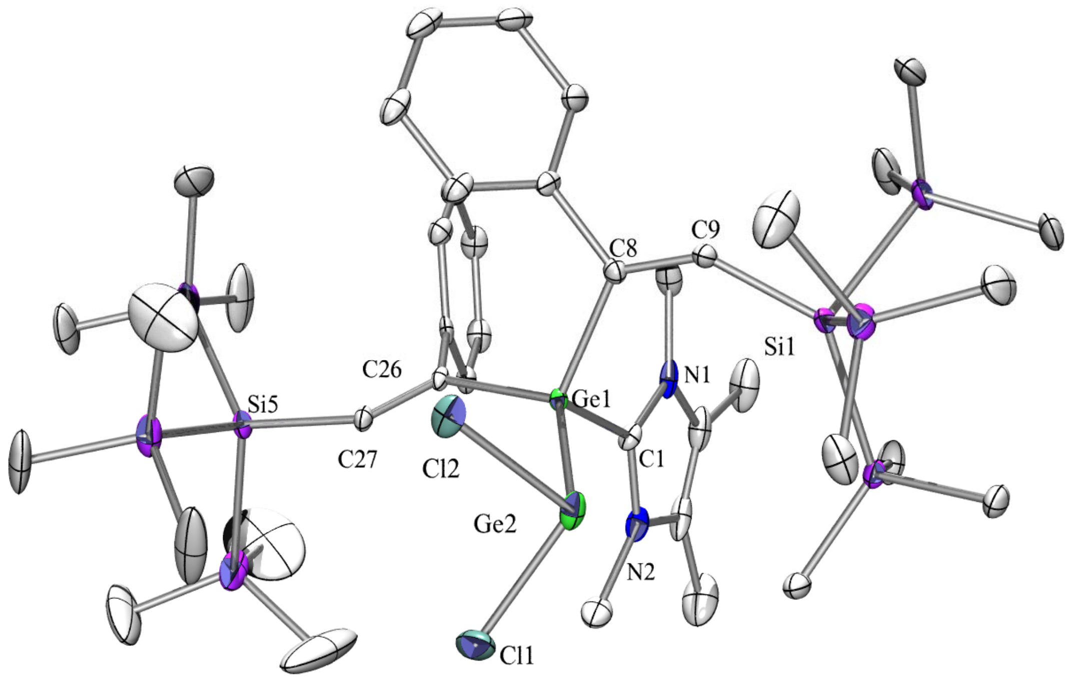

Figure 5. Molecular structure of **10** (thermal ellipsoid plot drawn at the 30% probability level, an additional THF molecule was found in the asymmetric unit). All hydrogen atoms are omitted for clarity (bond lengths in Å, angles in deg). Ge(1)-C(26) 1.995(4), Ge(1)-C(8) 1.999(4), Ge(1)-C(1) 2.006(4), Ge(1)-Ge(2) 2.5628(8), Ge(2)-Cl(2) 2.2997(14), N(1)-C(1) 1.354(6), Si(1)-C(9) 1.890(5), Si(1)-Si(2) 2.3539(19), Si(5)-C(27) 1.897(4), C(8)-C(9) 1.344(6), C(26)-C(27) 1.336(6), C(26)-Ge(1)-C(8) 111.56(17), C(26)-Ge(1)-C(1) 96.80(17), C(8)-Ge(1)-C(1) 105.12(18), C(26)-Ge(1)-Ge(2) 122.36(12), C(8)-Ge(1)-Ge(2) 101.24(13), C(1)-Ge(1)-Ge(2) 119.04(13), N(2)-C(1)-N(1) 105.9(4), C(8)-C(9)-Si(1) 143.6(4), C(26)-C(27)-Si(5) 132.7(3).

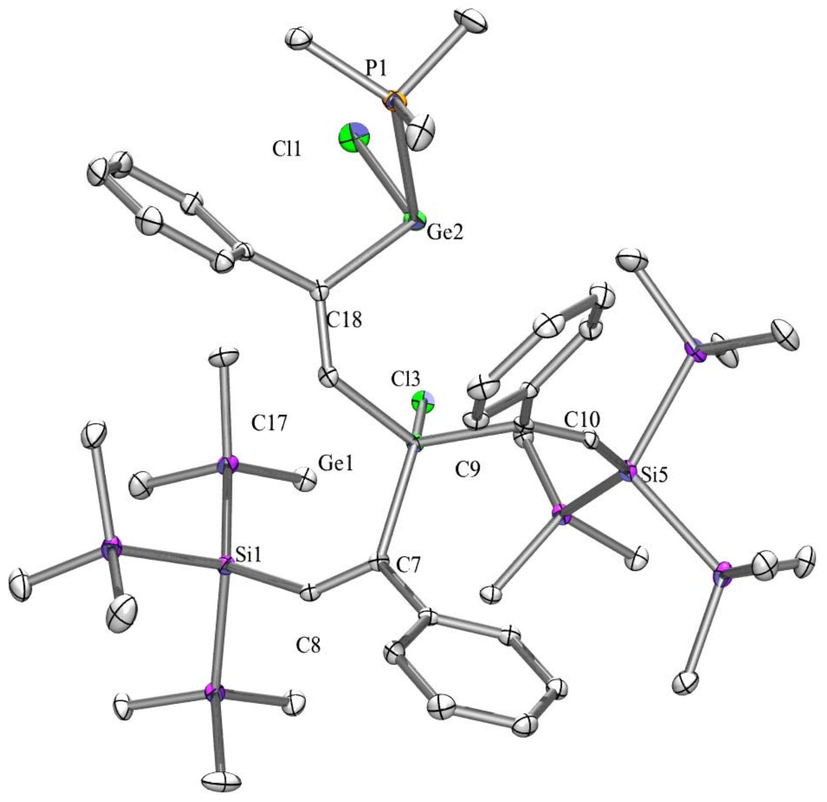

Figure 6. Molecular structure of **11** (thermal ellipsoid plot drawn at the 30% probability level). All hydrogen atoms are omitted for clarity (bond lengths in Å, angles in deg). Ge(1)-C(17) 1.942(3), Ge(1)-C(9) 1.961(3), Ge(1)-C(7) 1.963(3), Ge(1)-Cl(3) 2.1662(9), Ge(2)-C(18) 2.031(3), Ge(2)-Cl(1) 2.2962(11), Ge(2)-P(1) 2.4306(10), P(1)-C(43) 1.802(4), Si(1)-C(8) 1.905(3), Si(1)-Si(2) 2.3654(14), Si(5)-C(10) 1.897(3), C(7)-C(8) 1.340(4), C(9)-C(10) 1.329(4), C(17)-C(18) 1.327(4), C(17)-Ge(1)-C(9) 116.70(13), C(17)-Ge(1)-C(7) 104.41(13), C(9)-Ge(1)-C(7) 113.29(13), C(7)-C(8)-Si(1) 142.8(3), C(9)-C(10)-Si(5) 144.3(3).

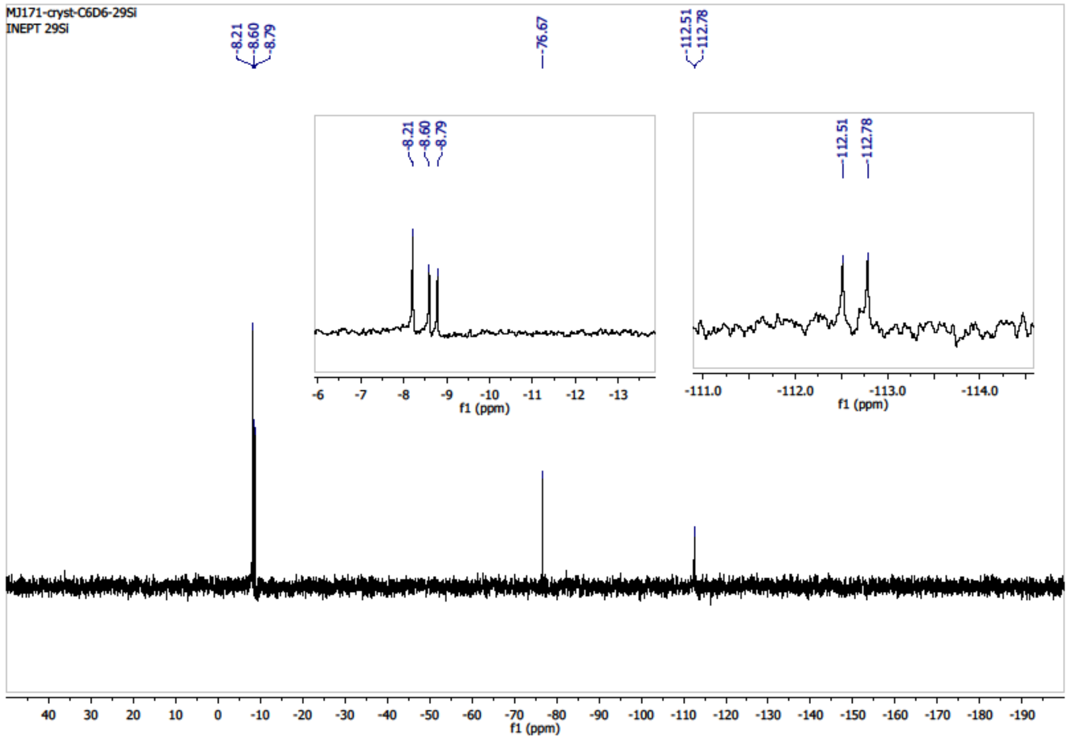

Figure 7. $^{29}Si\{H\}$ INEPT NMR spectrum of **3** in C_6D_6

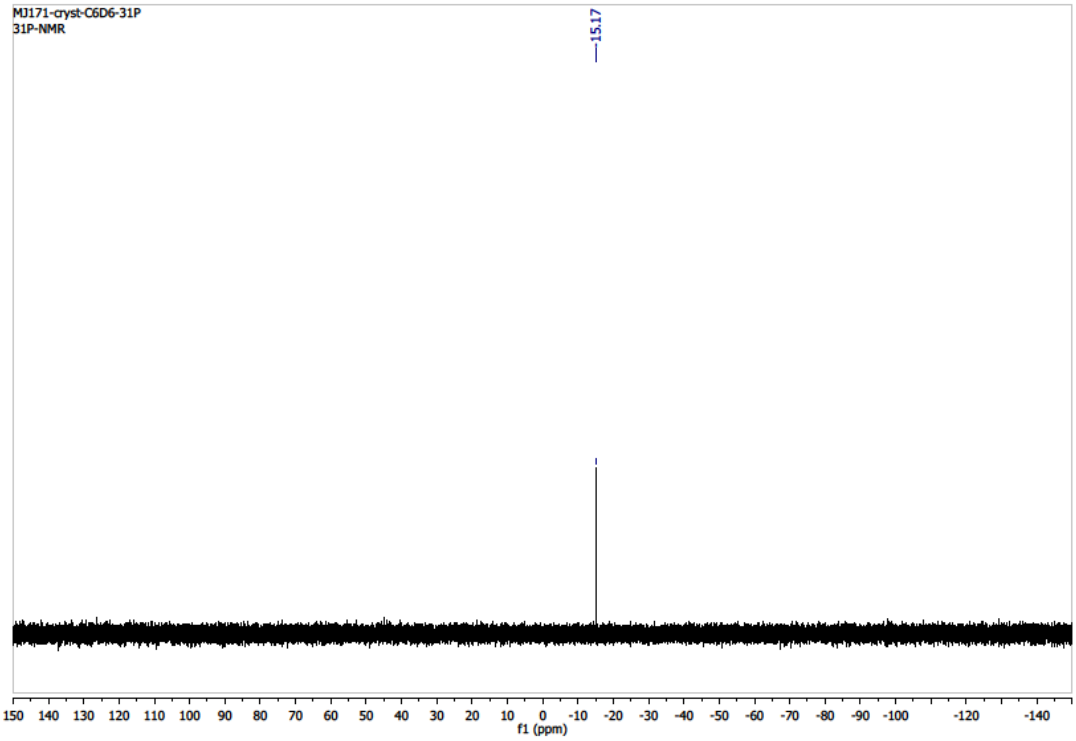

Figure 8. $^{31}P\{H\}$ NMR spectrum of **3** in C_6D_6

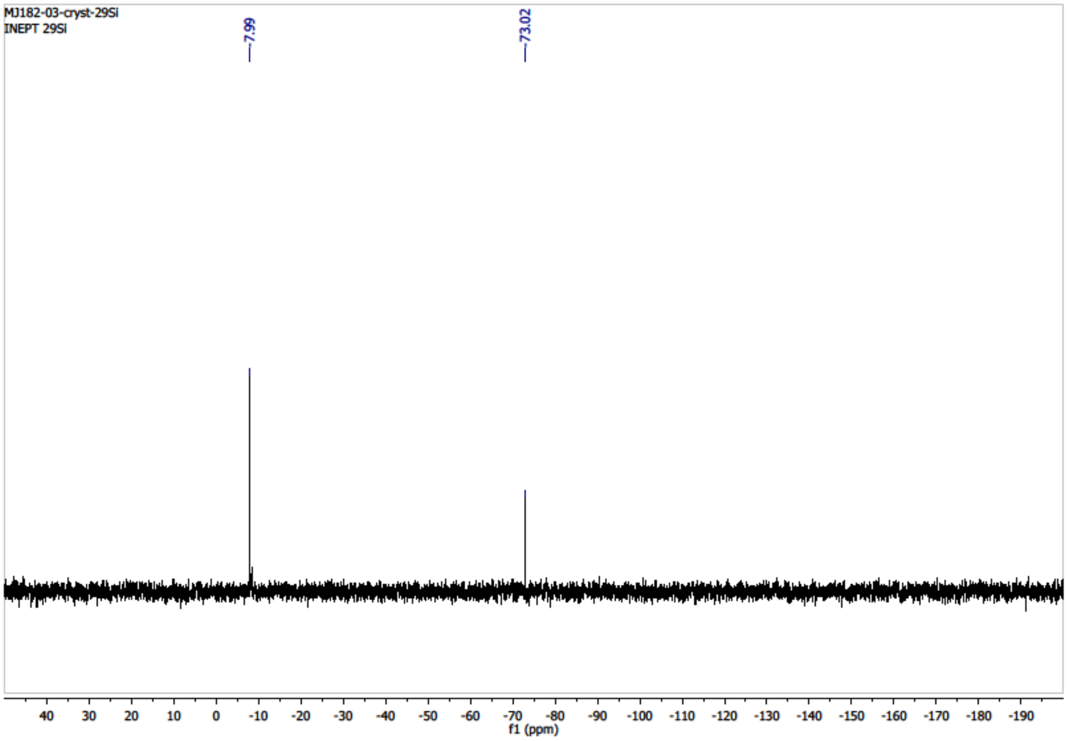

Figure 9. $^{29}Si\{H\}$ INEPT NMR spectrum of **4** in C_6D_6

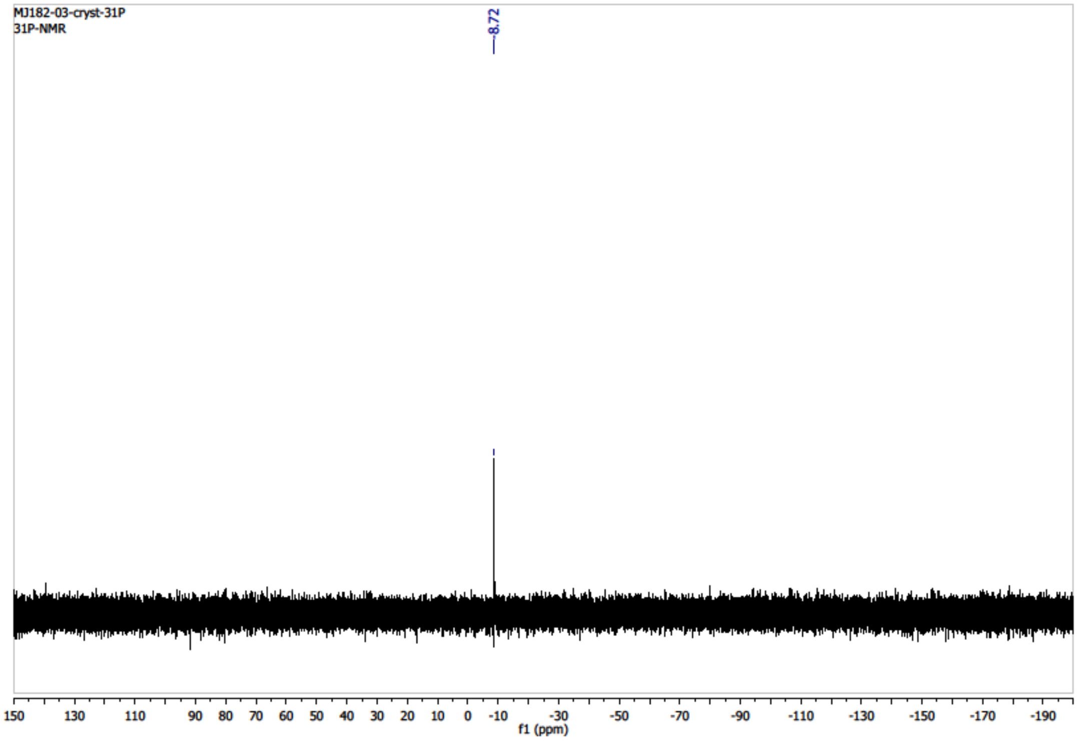

Figure 10. $^{31}P\{H\}$ NMR spectrum of **4** in C_6D_6

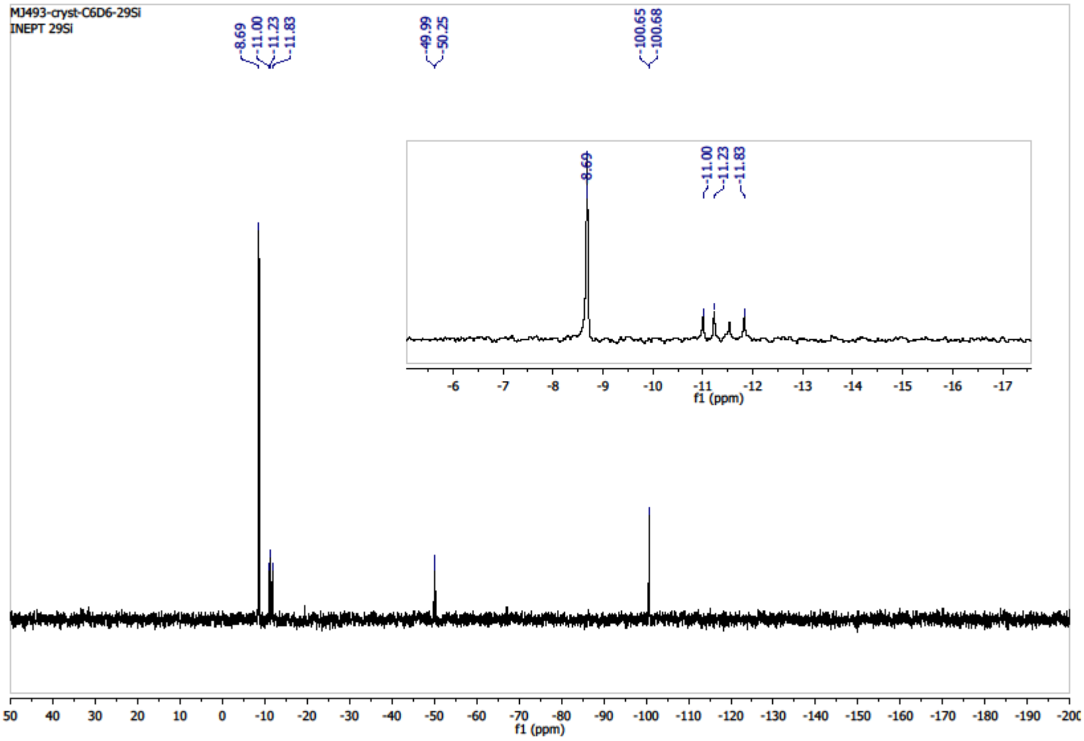

Figure 11. $^{29}Si\{H\}$ INEPT NMR spectrum of **5** in C_6D_6

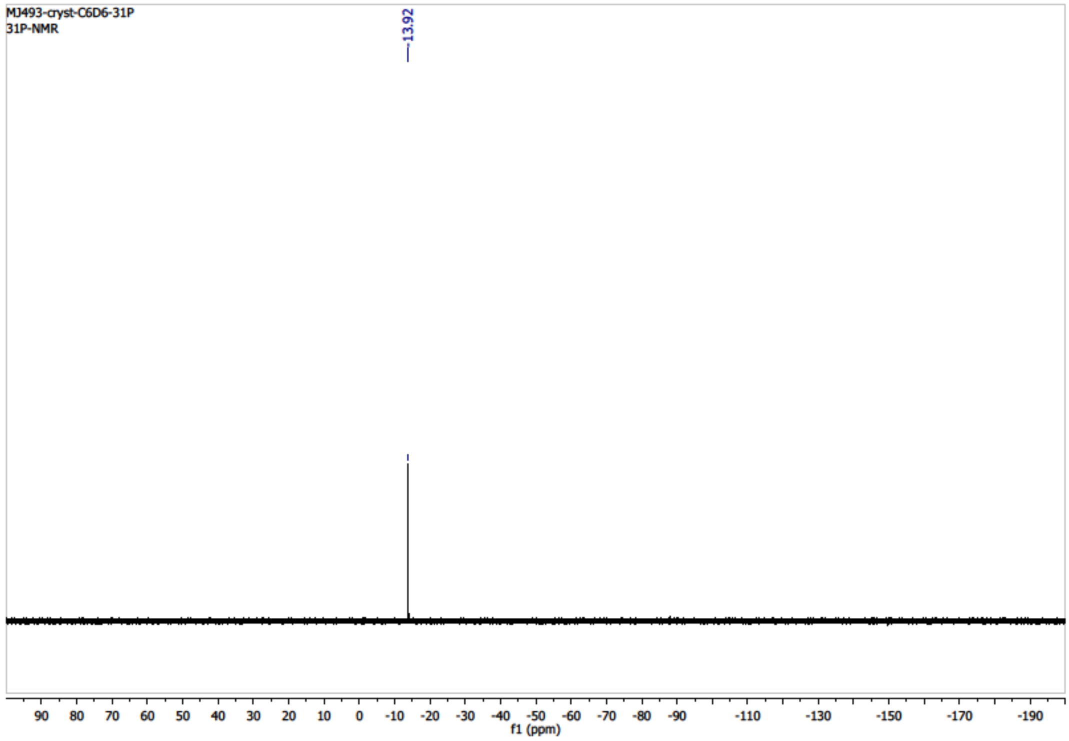

Figure 12. $^{31}P\{H\}$ NMR spectrum of **5** in C_6D_6

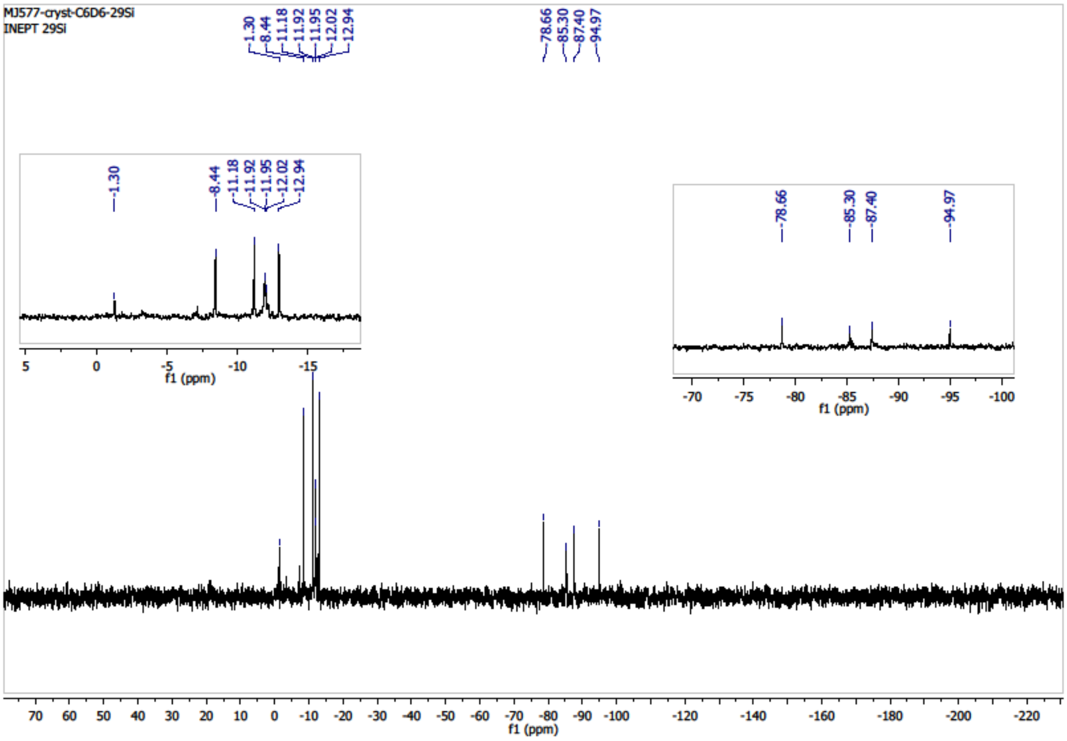

Figure 13. $^{29}Si\{H\}$ INEPT NMR spectrum of **7**/**8a** in C_6D_6

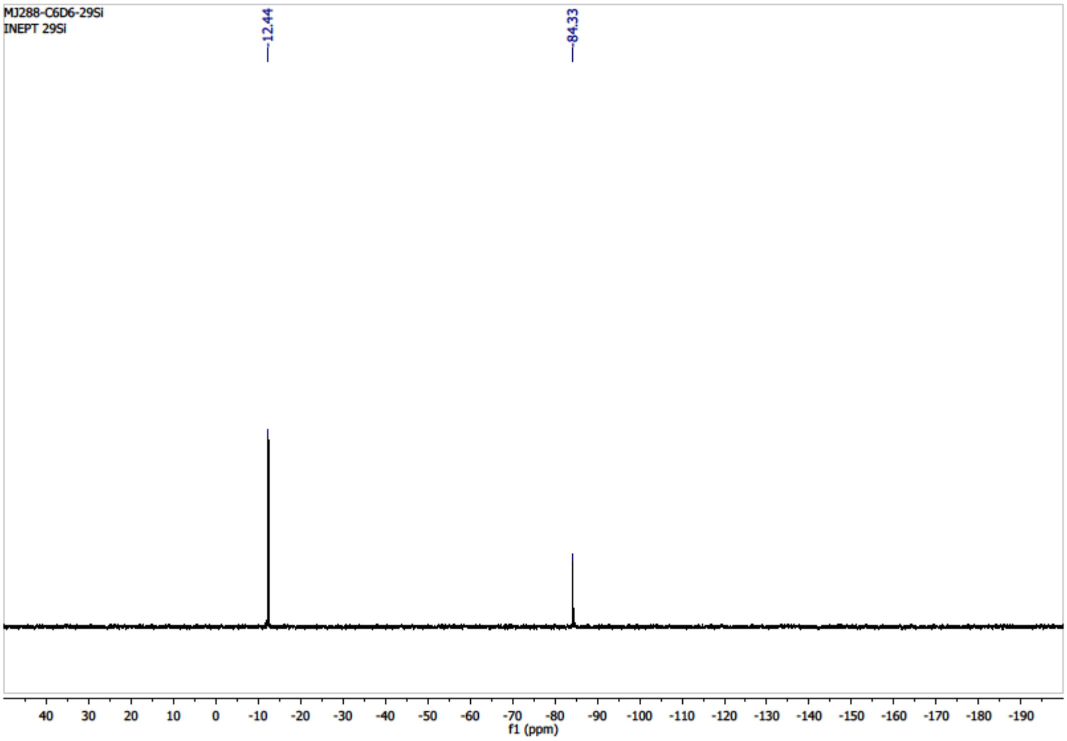

Figure 14. $^{29}Si\{H\}$ INEPT NMR spectrum of **10** in C_6D_6
